# Double‐ to Single‐Strand Transition Induces Forces and Motion in DNA Origami Nanostructures

**DOI:** 10.1002/adma.202101986

**Published:** 2021-08-01

**Authors:** Fatih N. Gür, Susanne Kempter, Florian Schueder, Christoph Sikeler, Maximilian J. Urban, Ralf Jungmann, Philipp C. Nickels, Tim Liedl

**Affiliations:** ^1^ Faculty of Physics and Center for NanoScience (CeNS) Ludwig‐Maximilians‐University Geschwister‐Scholl‐Platz 1 80539 Munich Germany; ^2^ Research Group Molecular Imaging and Bionanotechnology Max Planck Institute of Biochemistry Am Klopferspitz 18 82152 Martinsried Germany; ^3^ Department of Cell Biology Yale University School of Medicine 333 Cedar Street New Haven 06510 United States

**Keywords:** DNA origami nanostructures, DNA‐PAINT, entropic force, nanoparticles, single‐stranded DNA, super‐resolution microscopy

## Abstract

The design of dynamic, reconfigurable devices is crucial for the bottom‐up construction of artificial biological systems. DNA can be used as an engineering material for the de‐novo design of such dynamic devices. A self‐assembled DNA origami switch is presented that uses the transition from double‐ to single‐stranded DNA and vice versa to create and annihilate an entropic force that drives a reversible conformational change inside the switch. It is distinctively demonstrated that a DNA single‐strand that is extended with 0.34 nm per nucleotide – the extension this very strand has in the double‐stranded configuration – exerts a contractive force on its ends leading to large‐scale motion. The operation of this type of switch is demonstrated via transmission electron microscopy, DNA‐PAINT super‐resolution microscopy and darkfield microscopy. The work illustrates the intricate and sometimes counter‐intuitive forces that act in nanoscale physical systems that operate in fluids.

## Graphical abstract

A designed pulling mechanism making use of the entropic spring behavior of single‐stranded DNA is presented. Controlled and reversible motion of two arms of a nanoscale DNA origami structure is shown, resembling the arms of a jumping jack. The opening and closing of the arms are monitored with transmission electron microscopy, super‐resolution microscopy, and dark‐field analysis.

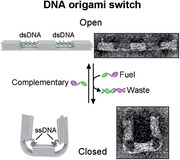

## 1 Introduction

Due to its predictable Watson‐Crick base‐pairing, DNA has been used successfully over the last decades as an engineering material for the bottom‐up self‐assembly of well‐defined structures and devices on the nanoscale.^[^ ^1^, ^2^, ^3^ ^]^ In particular, DNA origami,^[^ ^4^, ^5^, ^6^, ^7^ ^]^ in which a long single‐stranded DNA (ssDNA) scaffold is self‐assembled into 2D and 3D predefined shapes with a set of specifically designed short oligonucleotide staple‐strands, allows building unprecedented complex and functional nanostructures with high yields.^[^ ^8^ ^]^ DNA also adapts to mechanically stressed conformations, enabling the realization of curved, twisted, and bent DNA origami structures.^[^ ^9^, ^10^, ^11^ ^]^ By using the entropic spring behavior of ssDNA, the construction of prestressed tensegrity,^[^ ^12^ ^]^ bent,^[^ ^13^, ^14^ ^]^ and force clamping^[^ ^15^ ^]^ DNA structures has been reported. In related approaches, complementing the ssDNA gap regions in DNA origami trusses with the help of DNA polymerases resulted in unidirectional transitions from bent to straight trusses,^[^ ^16^ ^]^ or the formation of a rigidified tetrahedron through RecA protein filament assembly on ssDNA sections of a DNA origami tripod structure.^[^ ^17^ ^]^

Aside from static nanoarchitectures, DNA nanotechnology also enables the construction of dynamic and autonomous switches.^[^ ^18^ ^]^ The operation of these dynamic switches can be divided into two main categories: first, operation via molecular interaction and second, operation via external stimuli. The main molecular interactions employed to control motion on the nanoscale are DNA hybridization (mainly toehold‐mediated strand displacement) and base stacking. Examples of such motion controlled by molecular interactions include reconfigurable plasmonic devices,^[^ ^19^ ^]^ hinges,^[^ ^20^, ^21^ ^]^ tweezers,^[^ ^18^, ^22^ ^]^ rotary devices,^[^ ^23^, ^24^, ^25^, ^26^ ^]^ walkers,^[^ ^27^ ^]^ drug carriers^[^ ^28^, ^29^ ^]^ and robots sorting molecules or nanoparticles.^[^ ^30^, ^31^ ^]^ Other molecular interactions as driving mechanisms include target molecule binding^[^ ^32^, ^33^ ^]^ and aptamer^[^ ^28^, ^29^ ^]^ as well as nucleosome interactions.^[^ ^34^ ^]^ Operation via any molecular interaction, which includes all mechanism described above, has the advantage of controllable molecular recognition and specificity. However, the operation speed is limited by diffusion and interaction kinetics of the molecules and thus often quite slow. Notably, several approaches have been developed to increase the response speed of dynamic DNA devices. On the other hand, external stimuli such as light,^[^ ^35^, ^36^ ^]^ temperature,^[^ ^37^ ^]^ ions,^[^ ^11^, ^23^ ^]^ pH,^[^ ^38^, ^39^, ^40^ ^]^ and electric fields^[^ ^21^, ^41^ ^]^ often enable much faster operation up to an increase in speed by many orders of magnitude.^[^ ^41^ ^]^ For example, Karna et al. used the reversible, pH‐dependent formation of i‐motifs between adjacent nanostructure domains to facilitate the actuation of a coiled DNA nanospring that in turn impacts the motility of cultured cells via integrin coupling.^[^ ^40^ ^]^ Any of these stimuli which we here termed external, however, have the limitation of acting globally and they lack the specificity molecular recognitions can offer.

We here developed a molecular interaction‐based mechanism for the actuation of a DNA origami switch that performs against intuition to some extend: removing one of the two strands of a region of double‐stranded DNA (dsDNA) inside a DNA structure leaves a section of single‐stranded DNA (ssDNA) of the same length. This remaining single strand is now much floppier as the persistence length of ssDNA is ≈1 nm compared to ≈50 nm of dsDNA. This floppiness, however, does not lead to increased flexibility and extension but, on the contrary, to a substantial contraction of the region in question. This spring‐like behavior results primarily from the entropic properties of a polymer in solution that can theoretically be described using, for example, the modified freely jointed chain model (mFJC)^[^ ^42^ ^]^ or the worm‐like chain model (WLC).^[^ ^43^ ^]^ While this transition from dsDNA to ssDNA in our experiments is driven by strand‐displacement and thus suffers the same lack of reaction speed as previously described mechanisms, it offers great simplicity.

## 2 Results and Discussion

**Scheme 1:**
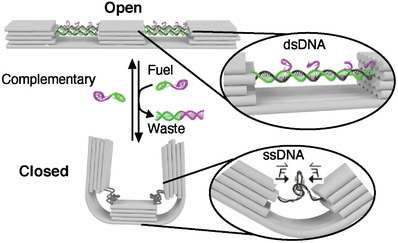
Schematic overview of the DNA origami switch. Close view of the dsDNA‐to‐ssDNA transition that leads to the reversible change between the open and closed state.

We designed a 140 nm long DNA origami switch composed of three rectangular blocks linked together by in total six parallel interconnected DNA double helices at the bottom (**Scheme** **1**). Four dsDNA helices (2 × 2) at the top, each formed by hybridization of four staple strands to the scaffold strand, interconnect the blocks and span the 87‐nucleotide (nt) ‐long (≈30 nm) gaps between the blocks. Each dsDNA bridge has four identical seven nt‐long toehold domains (violet) protruding from the 3’ ends of the staple strands (the design details and the list of oligonucleotides can be found in Figures S1–S3 and Table S2, Supporting Information). The transition from dsDNA to ssDNA occurs when an excess amount of fuel strands (complementary to the staple strands in the dsDNA bridge helices) are added. The strand displacement reaction^[^ ^22^, ^44^ ^]^ is initiated via the toehold domain and dislocates the staple strands from the scaffold, producing unreactive dsDNA waste. The reaction rate here is dependent on several parameters, including toehold length,^[^ ^45^ ^]^ overall strand length, concentration of added fuel strands, temperature and buffer conditions.^[^ ^46^ ^]^ In our system, with a 7 nt long toehold, a 22 nt long duplex length and a concentration of 100 × 10^−9^ m of the added fuel strand, the estimated response time for closing of the switch is on the order of seconds to minutes. To ensure efficient switching we thus incubated our switches for several hours. After the staple strand is removed, the entropic force of the remaining ssDNA now pulls the rectangular blocks toward each other at their upper part while bending the layer of double helices at the bottom of the DNA origami, facilitating a motion and change of state. As a rule of thumb one stretch of ssDNA that is extended to the length of its double‐stranded counterpart exerts a contractile force of ≈5.5 pN on its ends, irrespective of its total length. In our design, pairs of DNA duplexes bridge the upper half of the origami structure resulting in a pulling force of about 11 pN acting on each of the three blocks (see Figure S2 and Text S1 in the Supporting Information for a detailed description). While we avoided notorious hairpins within the m13mp18 scaffold for the choice of our spring regions, we were ad hoc not able to find four regions that completely lack secondary structures. Thermodynamic analysis of the four regions enabled by the NUPACK suite^[^ ^47^ ^]^ reveals possible formations of short hairpin stems of up to 5 base pairs (bps) (Figure S4, Supporting Information). Such hairpins will be transient but may add residual forces to the springs.^[^ ^48^ ^]^ In the next step, the addition of an excess of the sequences that have been removed before leads to these staple strands hybridizing again to the ssDNA scaffold, realizing the reverse transition (from ssDNA to dsDNA). This re‐opening of the switch is again expected to occur on the minute time scale, again we incubated the samples for hours. The switching between the open and closed states can thus be controlled through a series of dissociation and hybridization steps.

**Figure 1:**
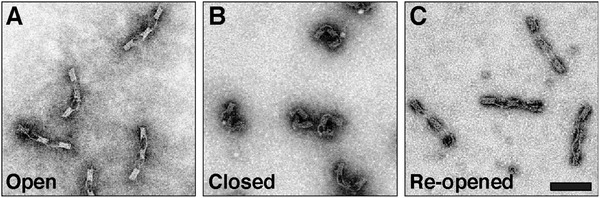
TEM micrographs of the DNA origami switch in the A) open, B) closed, and C) re‐opened states. Scale bar: 100 nm.

The DNA origami switch was self‐assembled in the open state in a one‐pot reaction by thermal annealing. Correct assembly of the switches was confirmed by agarose gel electrophoresis and transmission electron microscopy (TEM) (**Figure** **1** and Figure S5: Supporting Information). After folding and purification, an excess amount of fuel strands was added to the solution containing the samples to mediate the strand displacement and thus switch the device from the open to closed, U‐shaped state as apparent from the TEM micrographs shown in Figure 1B and Figures S6–S8 (Supporting Information). We measured the end‐to‐end distance of the switch in both states. The histogram of the end‐to‐end distance followed an asymmetric distribution skewed slightly toward smaller distances than designed for the open state and larger distances for the closed state (Figure S9, Supporting Information). Both distributions were approximated with a lognormal fit and the median end‐to‐end distance was calculated to be 130 nm in the open state and 33 nm in the closed state. To test the reversibility of our system, we further added an excess of staple strands complementary to the ssDNA scaffold region of the closed switches, leading to re‐opening (Figure 1C and Figure S8: Supporting Information). The distribution of the end‐to‐end distance after re‐opening was almost identical to the distribution of the initial open state with a median end‐to‐end distance of 130 nm (Figure S9: Supporting Information). It has to be noted, however, that this “perfect” result is partly an effect of TEM imaging being performed on dried samples. Structures that are slightly bent in solution will stretch out to full length when adsorbing with their bottom‐ or topsides on the TEM grid.

**Figure 2:**
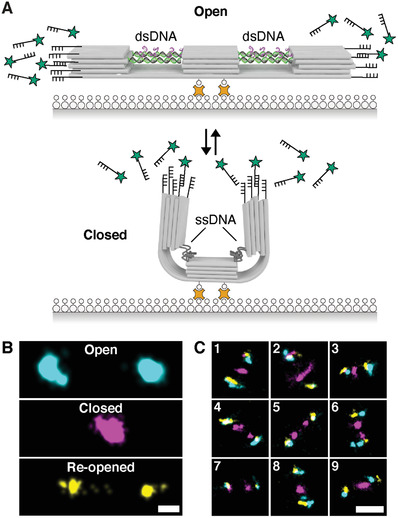
Observing switching of individual structures via DNA‐PAINT. A) Surface immobilization of the DNA origami switch on a glass substrate via biotin‐streptavidin conjugation. DNA origami structures carry DNA‐PAINT binding sites at both ends. B) DNA‐PAINT super‐resolution imaging to visualize different states of individual switches. Cyan represents the open state, magenta the closed state and yellow the re‐opened state. Scale bar: 25 nm. C) Overlay of the three consecutive imaging rounds of nine different switches. Colocalization of at least two of the three channels (cyan, magenta, and yellow) results in white spots. Scale bar: 100 nm.

To demonstrate the consecutive switching of individual DNA origami structures and to obtain information about configurational variability in solution, we employed multiplexed DNA‐PAINT super‐resolution microscopy.^[^ ^49^, ^50^ ^]^ First, we immobilized the structures (in the open state) via biotin‐modified DNA strands extending from the bottom under the central block to a BSA‐biotin coated glass surface (linked by streptavidin, **Figure** **2**A). In order to visualize the state of the DNA origami with DNA‐PAINT, we added docking sites on each end of the structure. In the first imaging round, we visualized the open state configuration. The measured center‐to‐center distance of the localization cluster of about 130 nm is in good agreement with the end‐to‐end distance measured in TEM micrographs (Figure 2B, cyan). To switch the DNA origami structure to its closed configuration, we incubated the origami with the fuel strands while being mounted on the microscope. The second round of DNA‐PAINT imaging depicts the closed state (Figure 2B, magenta). Next, we switched the structure back to the open state and performed a third round of imaging (Figure 2B, yellow). In the re‐opened state, the measured distances between both ends were in some instances reduced compared to the initial open state. This can be explained by incomplete transitions back to the double‐stranded form (e.g., one or several of the 16 required staple strands missing). Although the structure did not always switch back to the full ≈130 nm distance, the experiment demonstrates the repeated switching capability between the two states of individual switches followed over time (nine different, exemplary overlays of the three imaging rounds are shown in Figure 2C, a set of 186 individual switches together with a distance analysis is shown in Figures S10–S13, Supporting Information).

**Figure 3:**
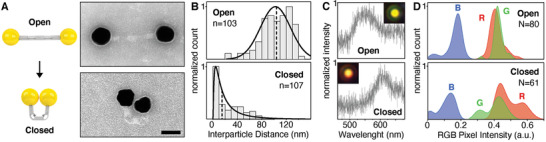
AuNP attachment to the DNA origami switches. A) Schematics showing the reversible switching between open and closed states of DNA structures carrying two AuNPs on opposing ends (left) and TEM micrographs of a representative structure for each state (right). Scale bar: 50 nm. B) Histograms of the interparticle distances (surface‐to‐surface) for the open state (top) and the closed state (bottom). Lognormal fits are drawn as a solid black line and the median as a dashed line; median_open_ = 102 nm; median_closed_ = 15 nm; *n* is the number of analyzed single nanoswitches. C) Darkfield images (insets) and scattering spectra of exemplary single switches in the open state (top) and closed state (bottom). The proximity of the AuNPs in the closed state leads to plasmon coupling and hence a red‐shift of the scattered light peak. D) Density plot (Kernel density estimation) of the RGB pixel intensity distribution in darkfield microscopy images of all analyzed single particles for the open state (top) and closed state (bottom). *N* is the number of analyzed single switches.

Next, we tested whether the structure can still perform controlled switching when it carries large cargos. We attached 50 nm gold nanoparticles (AuNPs) to both ends of the structure (**Figure** **3**A). AuNPs were functionalized with thiolated poly‐T oligonucleotides complementary to extensions on both ends of the switch structure. To attach the AuNPs to the designated position on the DNA origami, the switches with open states were mixed with functionalized AuNPs and then separated from excess AuNPs as well as aggregates by agarose gel electrophoresis (Figure S14, Supporting Information). Successful attachment of AuNPs as well as switching from open to closed to re‐opened state was verified by TEM (Figure 3A and Figures S15–S17: Supporting Information).

Furthermore, we analyzed the interparticle distance between two attached AuNPs for the open and closed states of > 100 switches in TEM micrographs (Figure 3B). We again approximated both distributions with a lognormal fit and the median interparticle distance was calculated to be 102 nm in the open state and 15 nm in the closed state. Similar to the distribution of the end‐to‐end distance from the bare switches (Figure S9, Supporting Information), the distance distribution is asymmetric, especially in the closed state.

Another method to monitor the interparticle distance is to record the scattered light from the AuNPs in darkfield microscopy. The individual 50 nm AuNPs used here exhibit plasmon resonance in the visible range (≈550 nm) and the coupling of plasmons^[^ ^51^ ^]^ from particles in close proximity results in a shift of the scattered light toward longer wavelengths.^[^ ^52^ ^]^ This plasmon coupling is highly distance dependent^[^ ^51^ ^]^ and indeed we observed discernible red‐shifts in darkfield microscopy images upon switching of the structures from the open to the closed state. Figure 3C shows two exemplary images together with scattering spectra: the open structure carrying two separated AuNPs appears green whereas the two AuNPs in direct proximity on the closed device bring about orange spots as well as a pronounced red‐shift in the spectrum. We also analyzed all individual structures in our darkfield microscopy images via the pixel intensity of each spot in the three channels of the RGB color camera (images of all individual structures are shown in Figure S18, Supporting Information). This intensity distribution of the three channels is shown in Figure 3D. As expected, we observed unimodal intensity distributions for the open structures in all three channels. However, for the closed switches we observed bimodal intensity distributions in both the red and the green channel with an additional peak of higher intensity in red and correspondingly a new peak of lower intensity in green. This attests that the AuNPs of a large fraction of the switched structures came close enough to enable plasmon coupling strong enough to be discernible with the RGB color camera. At the same time, we did not expect to see efficient coupling at interparticle distances greater than ≈15 nm. Since approximately half of the closed switches exhibit a larger interparticle distance (median_closed_ = 15 nm, Figure 1B), the observed bimodal intensity distributions in the red and green channels of the closed switches are in good agreement with our TEM‐based measurements of the interparticle distance. Note, however, that in contrast to PAINT imaging both TEM and darkfield measurements were performed with dried samples which potentially leads to particles being pushed closer together due to drying and surface tension effects.

## 3 Conclusion

Here we presented a nanoscale, fully operational DNA origami switch. The controllable and reversible reconfiguration of the switch is driven by the transition from dsDNA to ssDNA and vice versa. The contractive force responsible for the switching is the result of merely a change in elasticity upon transition from dsDNA to ssDNA. This simple mechanism of dynamic reconfiguration is hardcoded directly into the building material of the switch itself and it has the potential to become an integral component for the development of synthetic biological machineries that mimic essential cellular behaviors such as membrane transformation and sculpting. Another potential application of our sequence‐specific signal transduction mechanism will be biosensing where pathogenic RNA or DNA sequences trigger mechanical changes that are easily detectable on the single‐structure level.

## 4 Experimental Section

### DNA Origami Design

The DNA origami switch was designed using Cadnano in the square lattice mode.^[^ ^53^, ^54^ ^]^ The structure (design schematics in Scheme 1 and Figures S1 and S2: Supporting Information) has a rectangular single layer base made up of 6 helices (L 140 nm × W 12 nm). Three rectangular columns of equal size (L 30 nm W 12 nm H 10 nm) protrude from the single layer base at regular intervals with a gap of 30 nm. The columns are four helices high and in the second helix from the top the scaffold crosses all columns twice. The square lattice layout generates 10.67 bp per turn instead of the native dsDNA twist of 10.5 bp per turn. To avoid an underwinding of the helices in the square lattice arrangement, the twist was corrected via insertion of five base pair deletions along the structure in each helix. This twist correction as well as the adoption of the designed geometry was verified with CanDo (Figure S3, Supporting Information).^[^ ^55^ ^]^ Switching the structure between the open and closed state was performed via toehold‐mediated strand displacement. For DNA‐PAINT and AuNP‐attachment both ends were modified with the corresponding ssDNA extensions (sequences can be found in Table S2: Supporting Information).

### Formation and Purification of DNA Origami Nanostructures

DNA origami structures were folded as follows: 10 × 10^−9^ m scaffold (p8634)^[^ ^5^ ^]^ and 100 × 10^−9^ m of a single‐stranded staple strands (Eurofins Genomics) were mixed in folding buffer (1 × TE, 14 × 10^−3^ m MgCl2). The mixture was heated up to 65 °C in a thermocycler (BioRad) and gradually cooled to 25 °C over the course of 16 h (Table S1, Supporting Information). The folded structures were separated from excess staple strands via gel electrophoreses (1 % w/v agarose run at 7 V cm^−1^ for 1.5 h in 1 × TAE, 11 × 10^−3^ m MgCl^2^, pre‐stained with SYBR safe, Thermofisher). The desired band was excised, and the DNA origami was squeezed out of the gel, having the gel between a glass slide and a piece of a parafilm. Alternatively, the nanostructures were purified two times via PEG purification as previously described.^[^ ^56^ ^]^

### Negative Staining with Uranyl Formate and TEM Imaging

The empty grid (formvar/carbon‐coated, 300 mesh Cu; Ted Pella, Inc) was glow discharged under Argon in a plasma cleaner (Binder) for 60 s. The purified DNA origami structures were immobilized on the freshly treated grids for 3 min, followed by a washing step and a 10 s staining step each with a drop of a 2 % w/v Uranyl formate solution (SPI supplies). The negative stained DNA origami structures were imaged using a JEM‐1011 (Jeol) transmission electron microscope operating at 80 kV.

### Switching Between Open and Closed State

After folding, the nanostructure has single‐stranded staple strands annealed to the ssDNA‐regions in the scaffold that span the gaps between the three blocks. These staples all carry the following toehold‐extension on the 3′‐end: 5′‐staple‐TGGTATT‐3.^[^ ^57^ ^]^ To switch the structure from the open to the closed state, these staple strands were removed via toehold‐mediated strand displacement. For the region, where the scaffold is routing between the 3 columns, 16 short single stranded DNA strands, with a complementary sequence to the scaffold, were extended with a not complementary part. All switching steps were performed with an initial DNA origami concentration of 10 × 10^−9^ m in reaction buffer (1 × TAE, 11 × 10^−3^ m MgCl^2^). For each switching event, the DNA origami structures were incubated for 24 h at 30 °C. To go from open‐ to closed‐state, a 10 x molar excess of displacement strands over DNA origami structures was added. For switching back to the open‐state, a 100 × molar excess of the initial staple‐strands was added.

### DNA‐PAINT

Sample preparation and imaging buffers A and B as well as the oxygen scavenging system PCA/PCD/Trolox were prepared as previously described.^[^ ^58^ ^]^ For sample preparation of Figure 2, an 8‐well µ‐Slide VI0.5 (ibidi) was used as sample flow chamber. First, 100 µL of biotin labeled bovine albumin (Sigma‐Aldrich) (1 mg mL^−1^, dissolved in buffer A) was flushed into the chamber and incubated for 5 min. The chamber was then washed with 500 µL of buffer A. 100 µL of streptavidin (Thermo Fisher) (0.5 mg mL^−1^, dissolved in buffer A) was then flushed through the chamber and allowed to bind for 5 min. After washing with 500 µL of buffer A and subsequently with 500 µL of buffer B, 100 µL of biotin labeled DNA structures (≈2 × 10^−9^ m) in buffer B were flushed into the chamber and incubated for 8 min. The chamber was washed with 500 µL of buffer B. Finally, 100 µL of the imager solution in the corresponding imaging buffer was flushed into the chamber. Fluorescence imaging was carried out on an inverted microscope (Nikon Instruments, Eclipse Ti2) with the Perfect Focus System, applying an objective‐type TIRF configuration with an oil‐immersion objective (Nikon Instruments, Apo SR TIRF 100 ×, NA 1.49, Oil). A 561 nm (MPB Communications Inc., 2 W, DPSS‐system) laser was used for excitation. The laser beam was passed through cleanup filters (Chroma Technology, ZET561/10) and coupled into the microscope objective using a beam splitter (Chroma Technology, ZT561rdc). Fluorescence light was spectrally filtered with an emission filter (Chroma Technology, ET600/50m and ET575lp) and imaged on a sCMOS camera (Andor, Zyla 4.2 Plus) without further magnification, resulting in an effective pixel size of 130 nm (after 2 × 2 binning). Images were acquired with an imager strand concentration of 5 × 10^−9^ m (P1‐Cy3B, 9nt, Metabion) in imaging buffer. Here, 15 000 frames were acquired at 200 ms exposure time. The readout bandwidth was set to 200 MHz. Laser power (at 561 nm) was set to 60 mW (measured at the BFP of the objective). This power corresponds to an intensity of ≈400 W cm^−2^ at the sample plane. After the image acquisition of the first round (DNA origami in an open state), the chamber was rinsed with buffer B and then incubated with the displacement strand (≈100 × 10^−9^ m in buffer B for ≈10 h) in order to switch to the closed state. Next, the buffer solution was replaced by imaging buffer containing 5 × 10^−9^ m of P1 imager (for second round of imaging). After imaging of the second round (DNA origami in the closed state) the sample was rinsed again with buffer B followed by an incubation with the initial staple strands (≈100 × 10^−9^ m in buffer B for ≈5 h) to switch the structure back to the open configuration. Finally, the buffer solution was changed back to imaging buffer containing 5 × 10^−9^ m of P1 imager (for the third round of imaging). Fitting of the raw data as well as all post processing (drift correction, alignment and rendering) was carried out using the Picasso software package.^[^ ^58^ ^]^

### Functionalization of AuNP

AuNPs (BBI International) were functionalized with 5′‐thiolated 19‐T single‐stranded DNA oligonucleotides (Biomers). 2 mL of a 50 nm AuNPs solution was centrifuged at 5000 g for 5 min. The supernatant was discarded and the pellet was resolved to a final concentration of 0,1 % w/v SDS and 20 × 10^−3^ m NaCl. After adding 20 µL of thiolated 19‐T single‐stranded DNA oligonucleotides, the solution was sonicated and vortexed for 1 min each and then frozen at −80 °C for 15 min. To remove the excess of AuNPs and to separate monomers from dimers a 0.7 % w/g agarose gel at 7 V cm^−1^ for 1.5 h was run in running buffer (1 × TAE, 11 × 10^−3^ m MgCl^2^).

### Conjugation of AuNP to DNA Origami Nanostructures

A ratio of 10:1 (AuNPs : DNA origami) was used to conjugate functionalized AuNPs to DNA origamis. The mixture was incubated for 1 h at room temperature and separated with a 0.7 % w/g agarose gel at 7 V cm^−1^ for 1.5 h in running buffer (1 x TAE, 11 × 10^−3^ m MgCl^2^).^[^ ^59^ ^]^ The desired band with one AuNP attached on each end of the DNA origami structure was excised.

### Darkfield Imaging

Glass slides were sonicated for 5 min with HellmanexIII (Sigma‐Aldrich), washed 5 x with double distilled water and sonicated for 5 min each with Acetone (Roth) and 2‐Propanol (Roth). Clean glass slides were blown dry with nitrogen and glow discharged under oxygen in a plasma cleaner (Binder) for 5 min. In order to locate the sample in the center of the glass slide a circle was drawn with a grease pencil on the glass slide. Open and closed state DNA origami structures with AuNPs attached (50 × 10^−12^ m) were deposited inside the grease circle for one minute, washed two times with 1 mL doubly distilled water and blown dry with nitrogen. Samples for Darkfield microscopy were imaged with a home‐built dark‐field setup in transmission mode using a 100 × air objective (Olympus) and an oil condenser (Olympus NA 1.4) with a 100 W halogen bulb as illumination source. Images were taken with 100 ms exposure time and 2 × binning on a color CMOS Camera (Thorlabs Kiralux CS895CU). The dark‐field scattering spectra were collected with the same home‐built dark‐field coupled to an Acton SP2300 spectrometer (Princeton Instruments). To analyze the RGB intensity values of individual nanostructures, the images were first thresholded and individual spots then automatically detected.

## Conflict of Interest

The authors declare no conflict of interest.

## Supporting information

Supporting Information

## Data Availability

The data that supports the findings of this study are available in the supplementary material of this article. Additional data (such as spectra source files) are available from the corresponding author upon request.
