# Supplementary material for: Double‐ to Single‐Strand Transition Induces Forces and Motion in DNA Origami Nanostructures
Source: Adv Mater. 2021 Aug 1;33(37):2101986. doi: 10.1002/adma.202101986 (PMC7611957; doi:10.1002/adma.202101986)
Supplement: Supplementary file 1 — Supporting Information [file ADMA-33-2101986-s001.pdf]

© 2021 Wiley-VCH GmbH

ADVANCED MATERIALS

Supporting Information

for *Adv. Mater.,* DOI: 10.1002/adma.202101986

Double- to Single-Strand Transition Induces Forces and Motion in DNA Origami Nanostructures

*Fatih N. Gür, Susanne Kempter, Florian Schueder, Christoph Sikeler, Maximilian J. Urban, Ralf Jungmann, Philipp C. Nickels, and Tim Liedl**

Copyright WILEY-VCH Verlag GmbH & Co. KGaA, 69469 Weinheim, Germany, 2018.

Supporting Information

Double- to Single-Strand Transition Induces Forces and Motion in DNA Origami nanostructures

*Fatih N. Gür[+], Susanne Kempter[+], Florian Schueder, Christoph Sikeler, Maximilian J. Urban, Ralf Jungmann, Philipp C. Nickels and Tim Liedl**

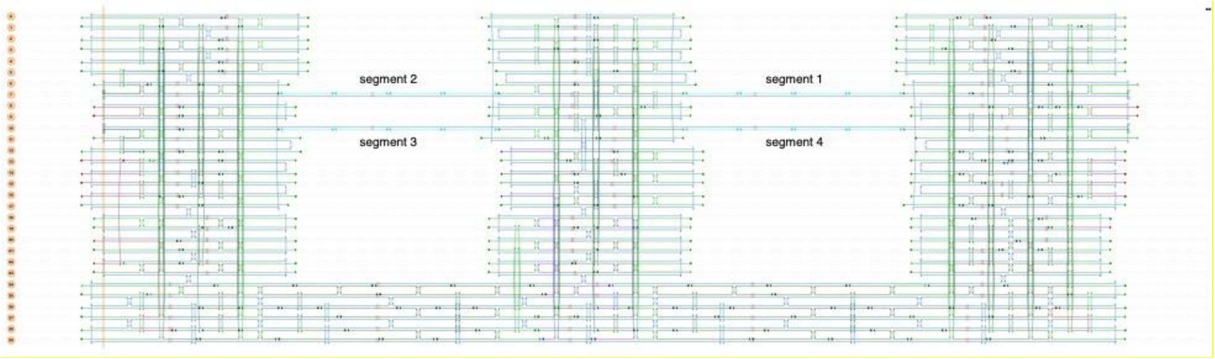

Figure S1. The CaDNAno design layout of the DNA origami switch. Blue: scaffold strand (p6834); green: core staple strands; red: handles for either DNA-PAINT or AuNPs attachment; purple: biotin attachment.

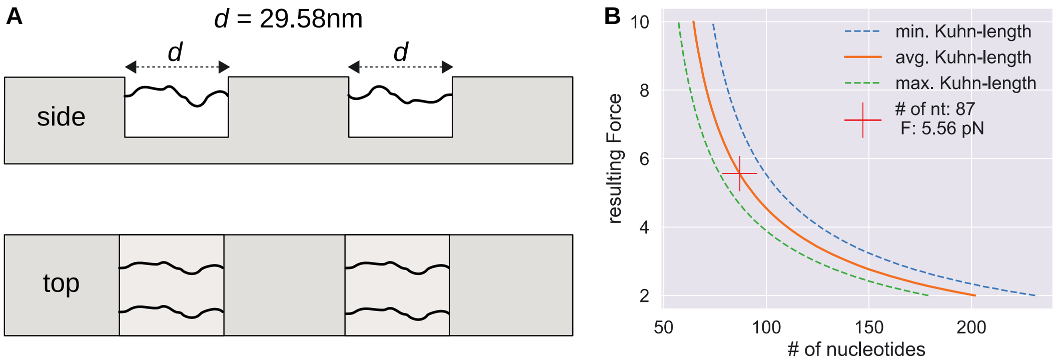

Figure S2. (A) Schematic drawing of the DNA origami switch design. (B) Resulting entropic force of each ssDNA-segment as a function of the number of nucleotides spanning the gap distance d.

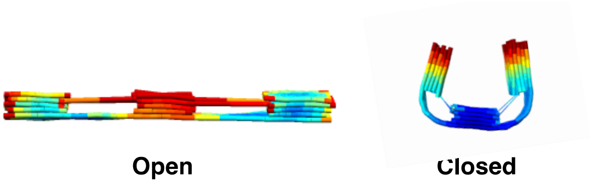

Figure S3. CanDo simulations of the switch design in the open and closed state.

Random permutations of the starting point of the M13-based, circular scaffold and the corresponding free energy of secondary structures in the four ssDNA-segments of these permutations:

	Segment 1 kcal/mol	Segment 2 kcal/mol	Segment 3 kcal/mol	Segment 4 kcal/mol
**1**	-4.74	-4.71	-8.60	-4.96
**2**	-11.73	-8.28	-7.54	-5.12
**3**	-10.04	-6.50	-11.11	-5.77
**4**	-14.34	-6.34	-3.67	-7.54

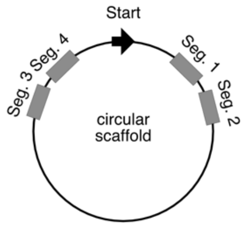

Predicted mean free energy of secondary structures for the sequences chosen from permutation #1:

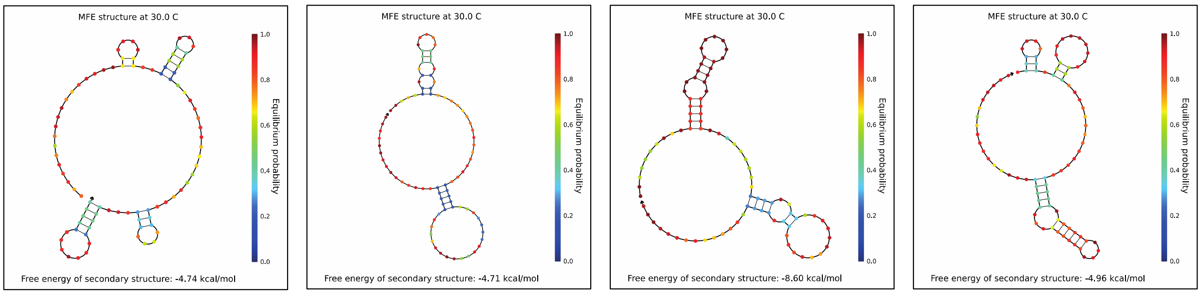

segment 1 segment 2 segment 3 segment 4

Predicted mean free energy of known hairpins in the M13 sequence as comparison:

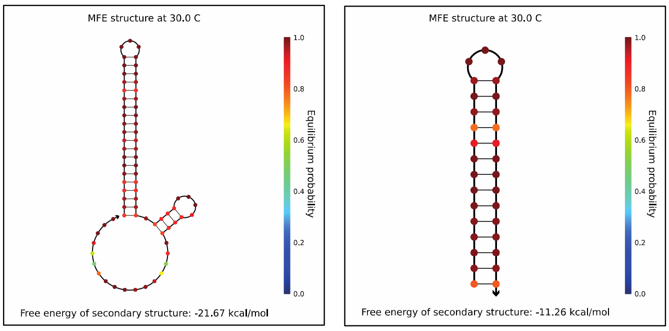

M13 - hairpin 1 M13 - hairpin 2

Figure S4. Thermodynamic NUPACK analysis of the ssDNA sections. **Top:** to minimize the effect of potential secondary structures, we performed four random permutations of the scaffold starting point and computed the mean free energy of occurring secondary structures inside the four segments. We then chose the permutation with the least predicted secondary structures (highlighted in green). **Middle:** the predicted mean free energy structures for the four selected ssDNA sequences. **Bottom:** mean free energy structure of two known hairpins in the M13mp18 sequence as comparison.

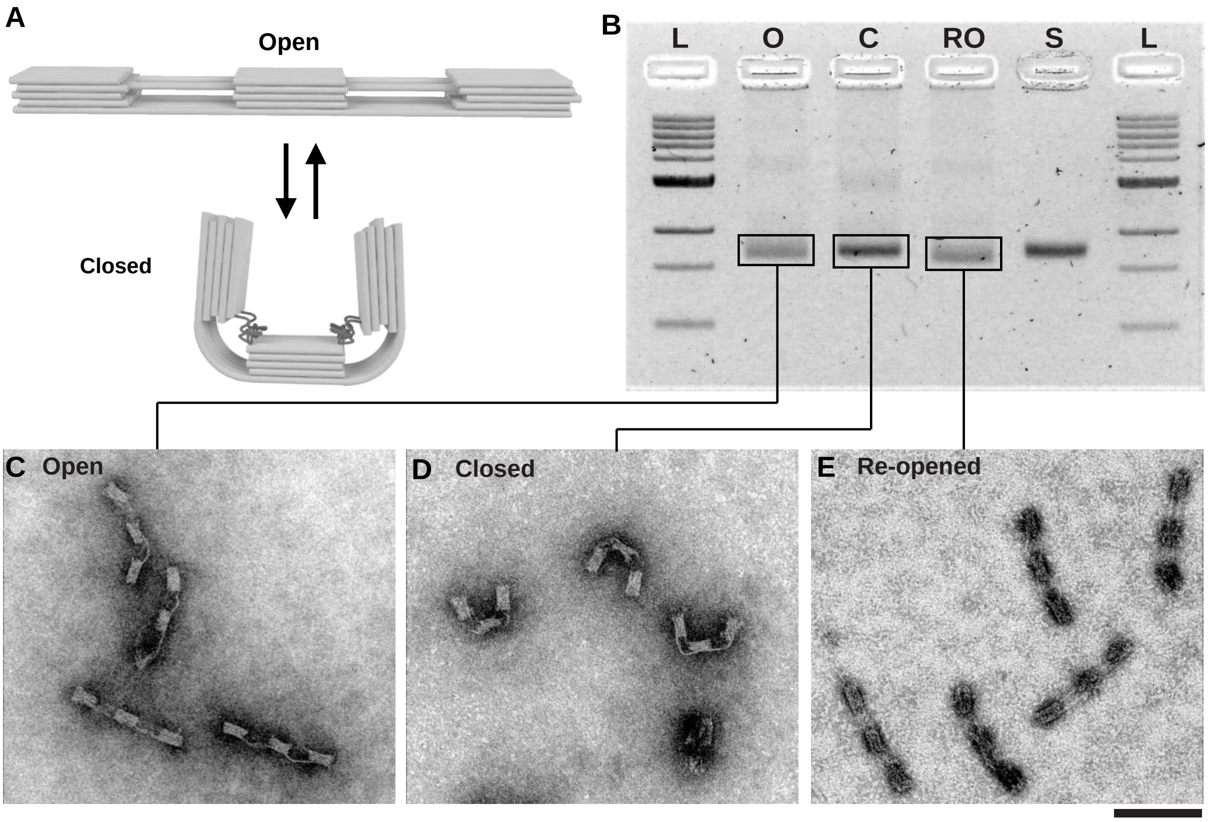

Figure S5. Agarose gel electrophoresis and TEM analysis of folded DNA origami switches. (A) Schematic representation of the DNA origami switch, illustrating open and closed states. (B) Agarose gel electrophoresis of DNA origami switch after folding and sequential switching from open to closed and re-opened states L: 1kb DNA ladder, O: open state, C: closed state, RO: re-opened state, S: p8634 scaffold. The bands containing correctly assembled structures were extracted and analyzed with TEM. TEM micrographs of the open (C), closed (D), and re-opened (e) states. Scale bar:100 nm.

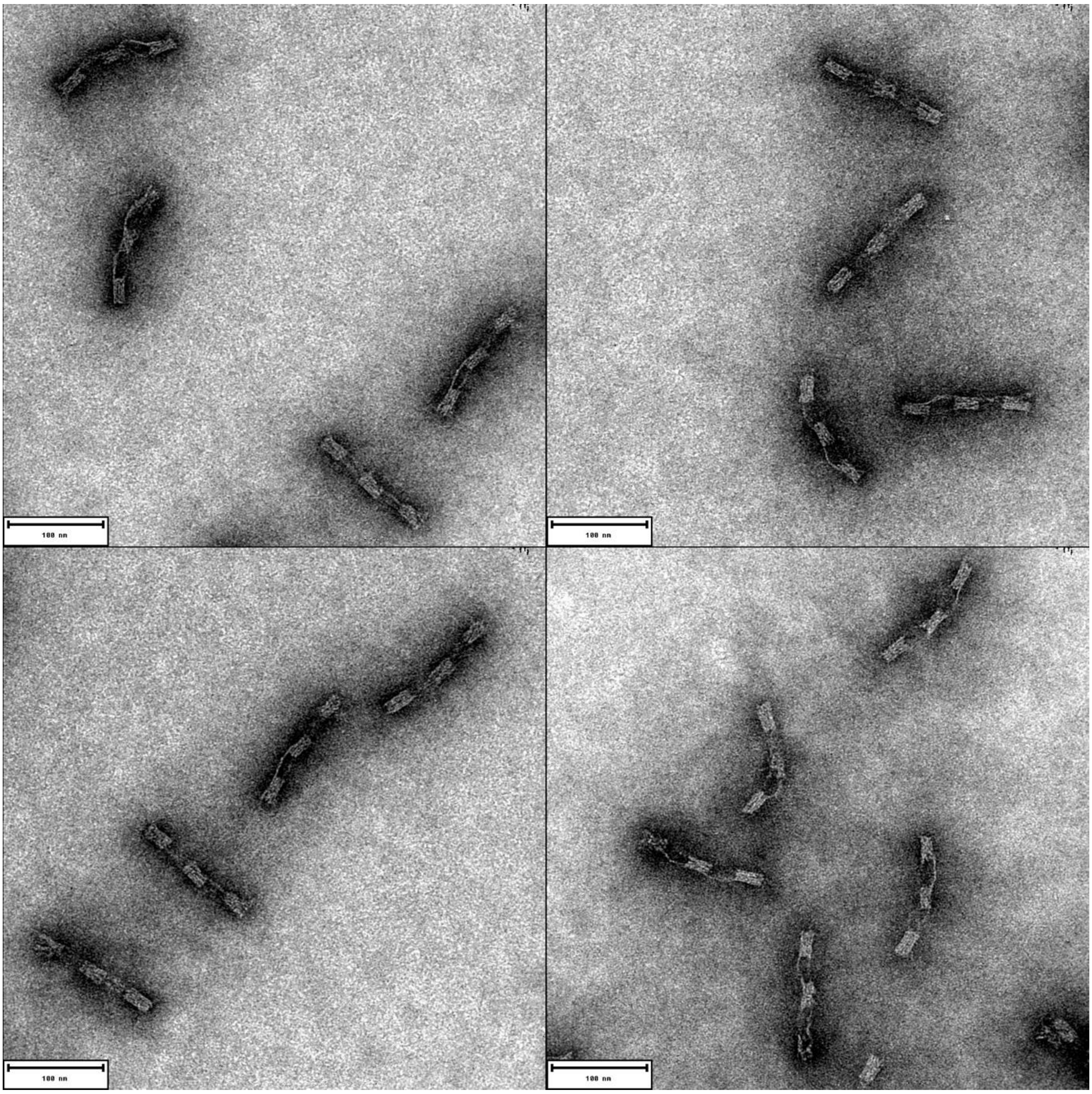

Figure S6. Exemplary TEM micrographs of the switch in the open state. Scale bars: 100 nm.

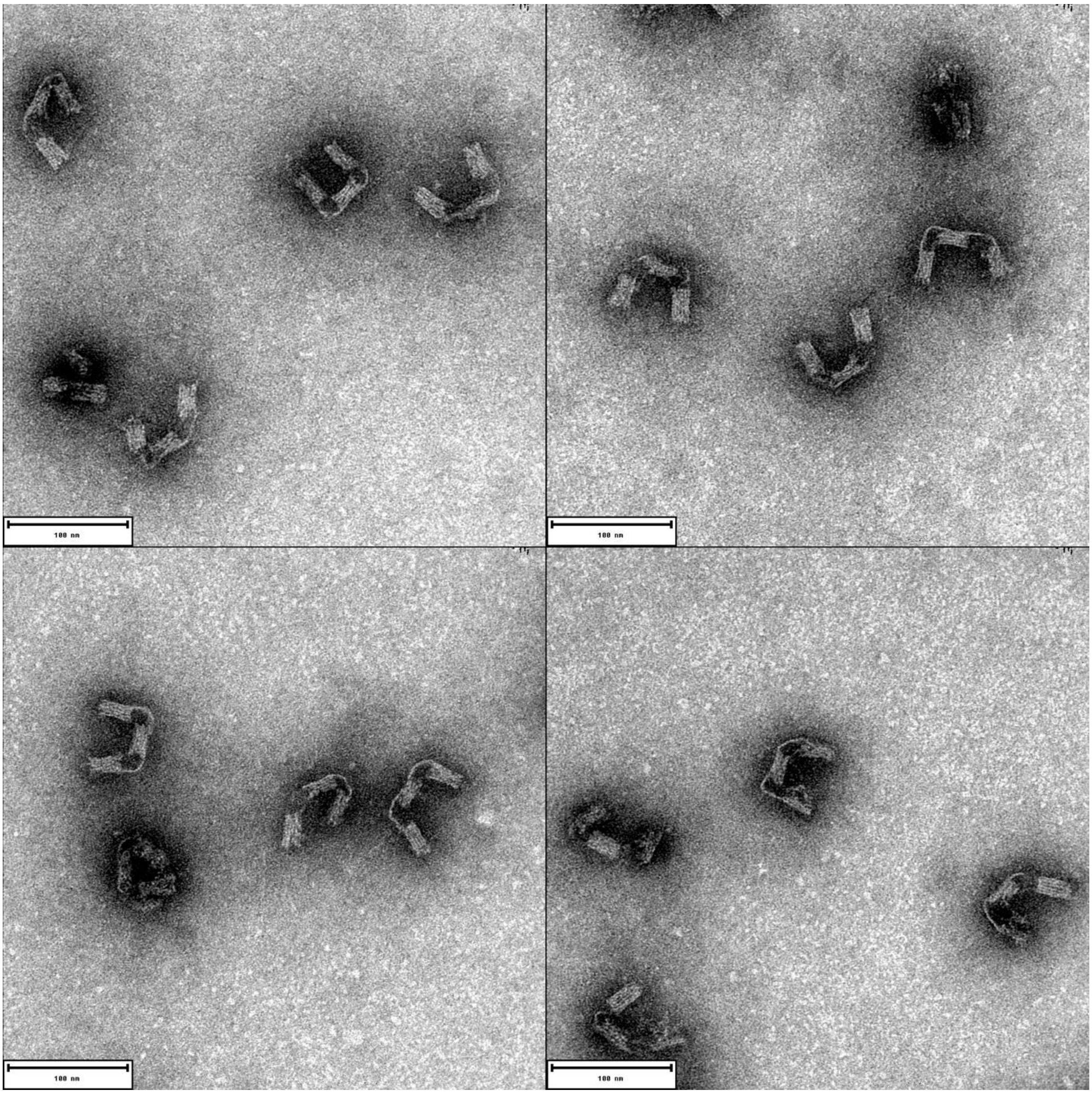

Figure S7. Exemplary TEM micrographs of the switch in the closed state. Scale bars: 100 nm.

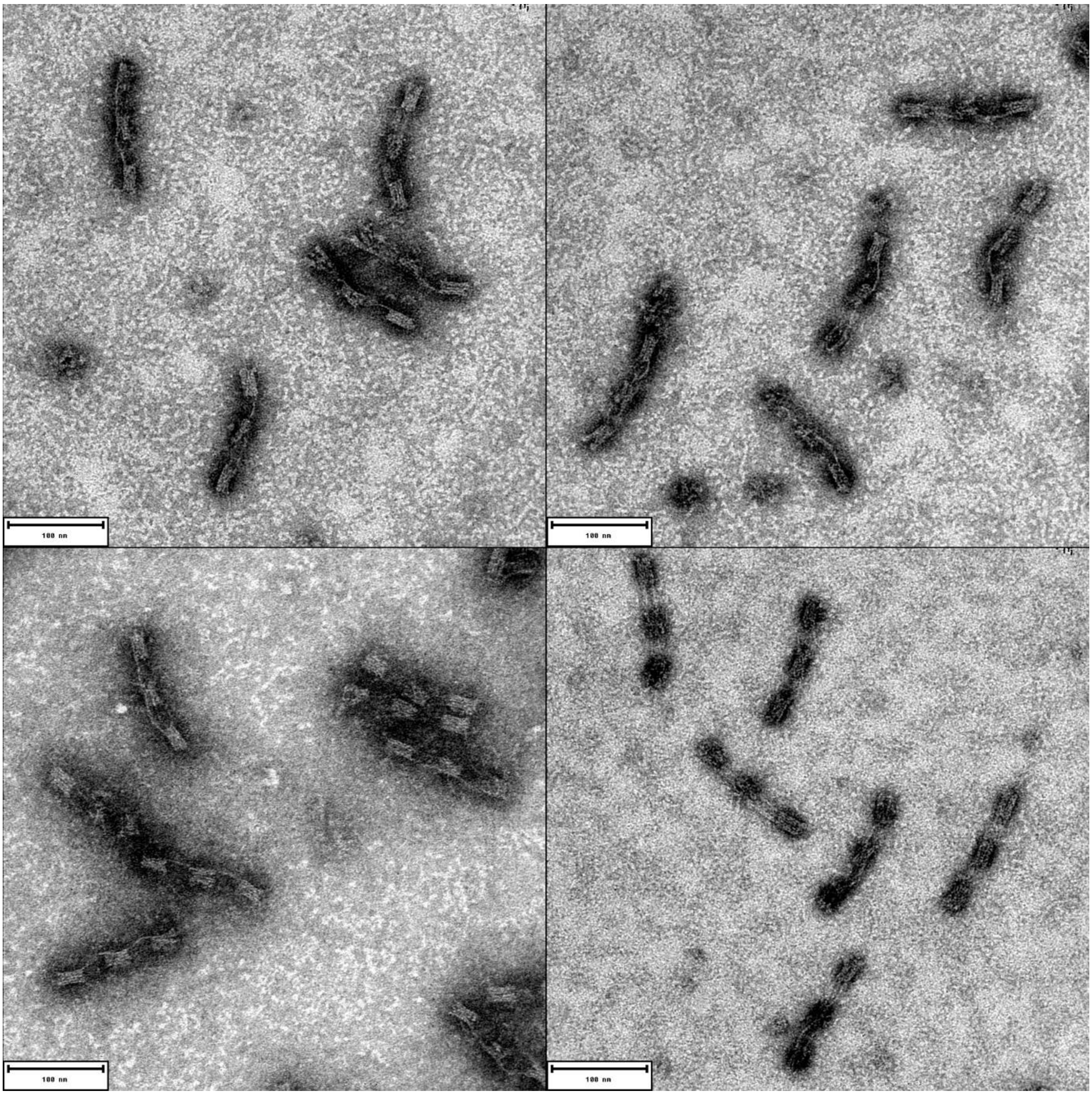

Figure S8. Exemplary TEM micrographs of the switch in the re-opened state. Scale bars: 100 nm.

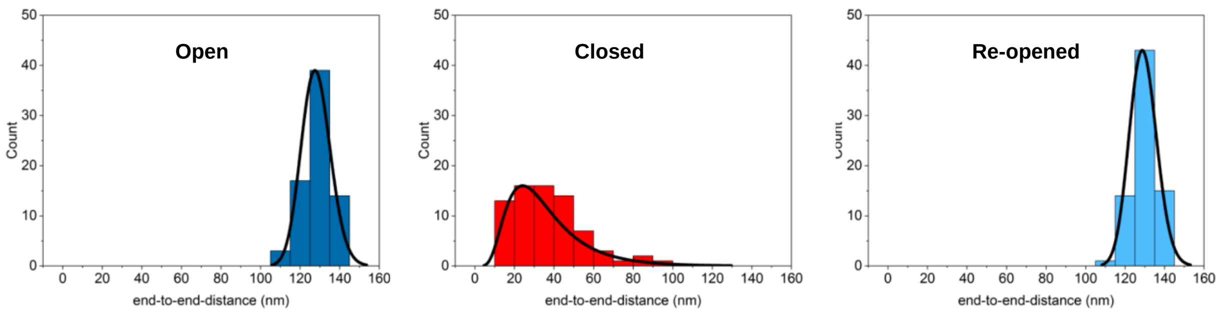

Figure S9. Distribution of the measured end-to-end distance of the switches in the open (left), closed (middle), and re-opened state (right). All distances were measured in TEM micrographs. Re-opening of the closed structures results in a distribution almost identical to the open state. All three distributions were fitted with a lognormal distribution (solid lines). $median_{open}$ = 130 nm; $median_{closed}$ = 33 nm; $median_{re\text{-}opened}$ = 130 nm.

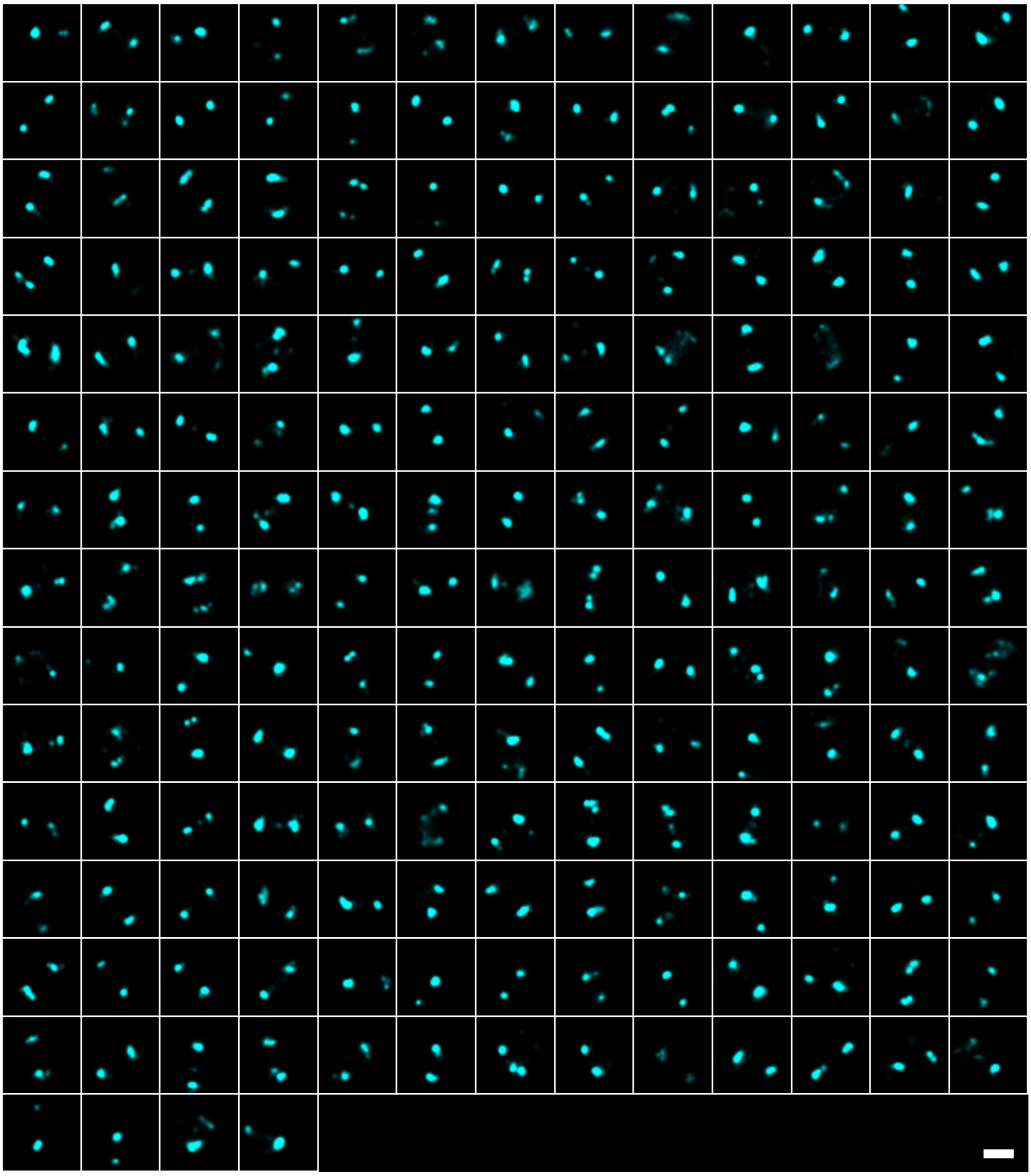

Figure S10. A set of 186 individual switches in the open conformation. Scale bar is 100 nm.

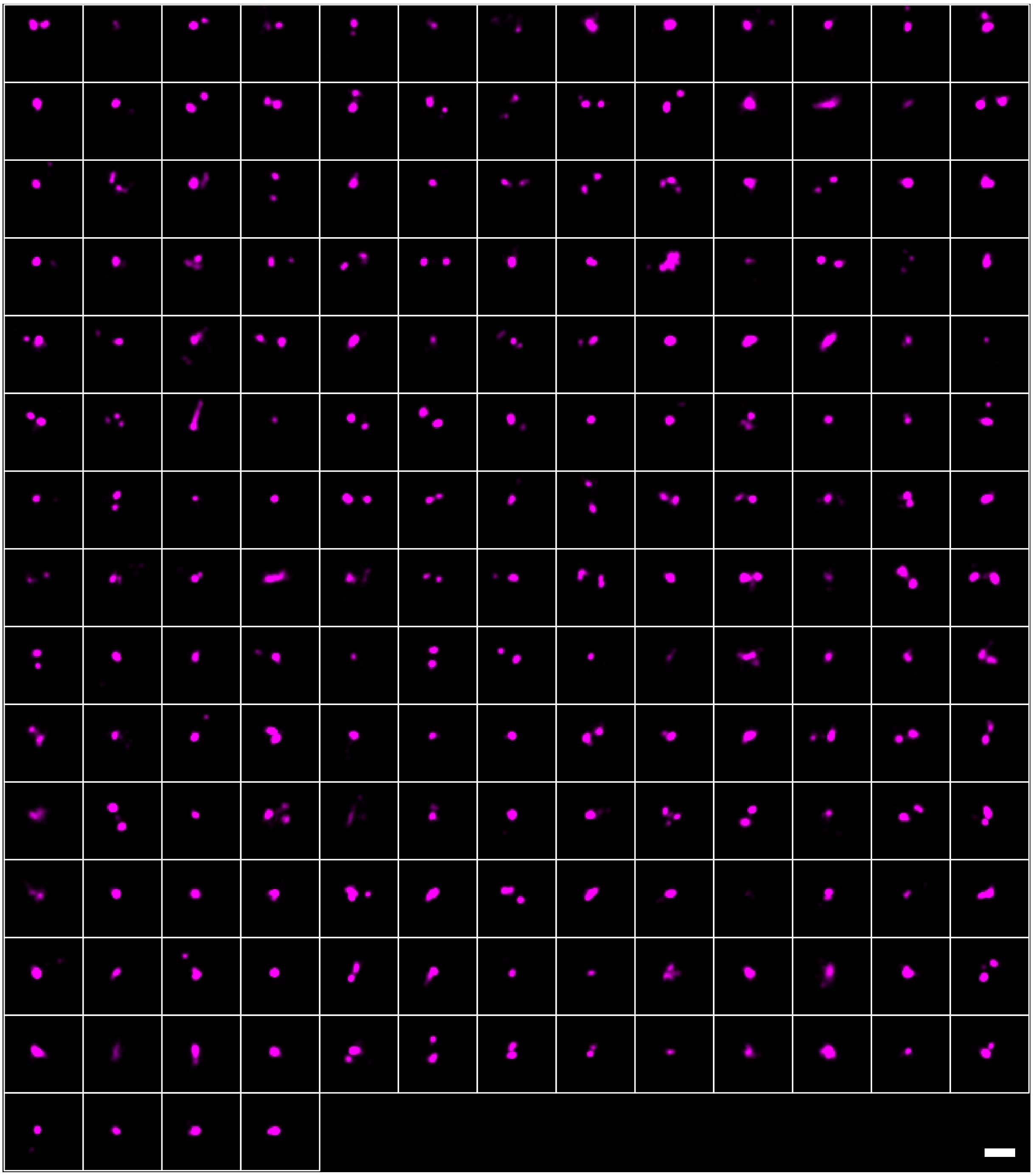

Figure S11. The same set of 186 individual switches after switching to the closed conformation. Scale bar is 100 nm.

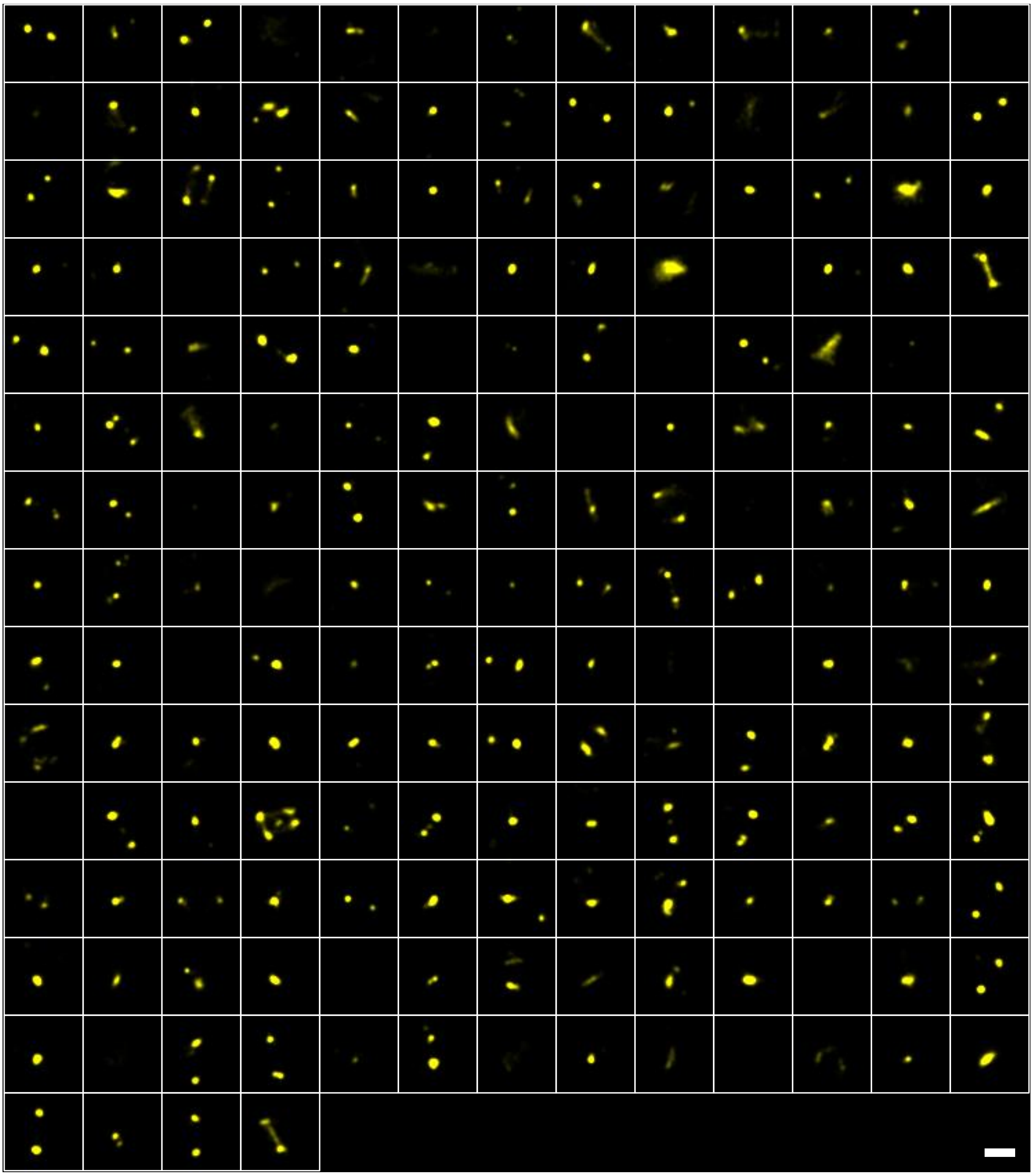

Figure S12. The same set of 186 individual switches after switching to the re-opened conformation. Scale bar is 100 nm.

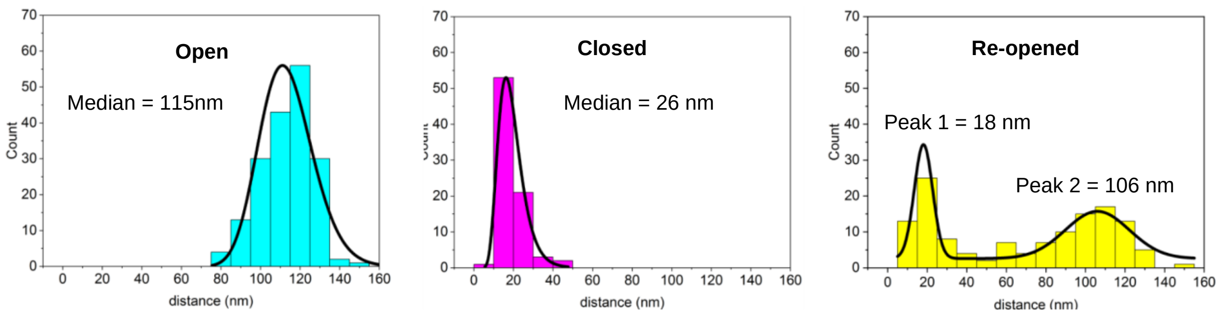

Figure S13. Distribution of the measured distance of the switches shown in figures S10-S12 in the open (left), closed (middle), and re-opened state (right). We computed the distances in the DNA-PAINT data by first fitting the localizations with either a bimodal gaussian distribution (for all switches with two distinct spots visible) or with a single gaussian (for all switches with only one spot visible). We then either measured the peak-to-peak distance of the bimodal gaussian distributions or took the FWHM (full width at half maximum) of the single gaussians as the distance value.

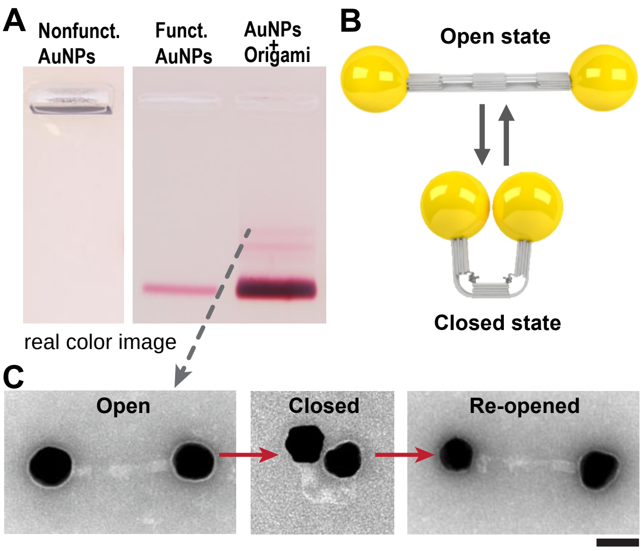

Figure S14. AuNP attachment to the switches. (A) Agarose gel electrophoresis for the purification of DNA origami switches carrying AuNPs from excess AuNPs. The band containing the switches with two AuNPs was cut and the correctly assembled structures were extracted. (B) Schematics showing reversible switching between open and closed states of the switches carrying two AuNPs (C) TEM analysis of open, closed, and re-opened states of the switches carrying two AuNPs. Scale bar: 50 nm.

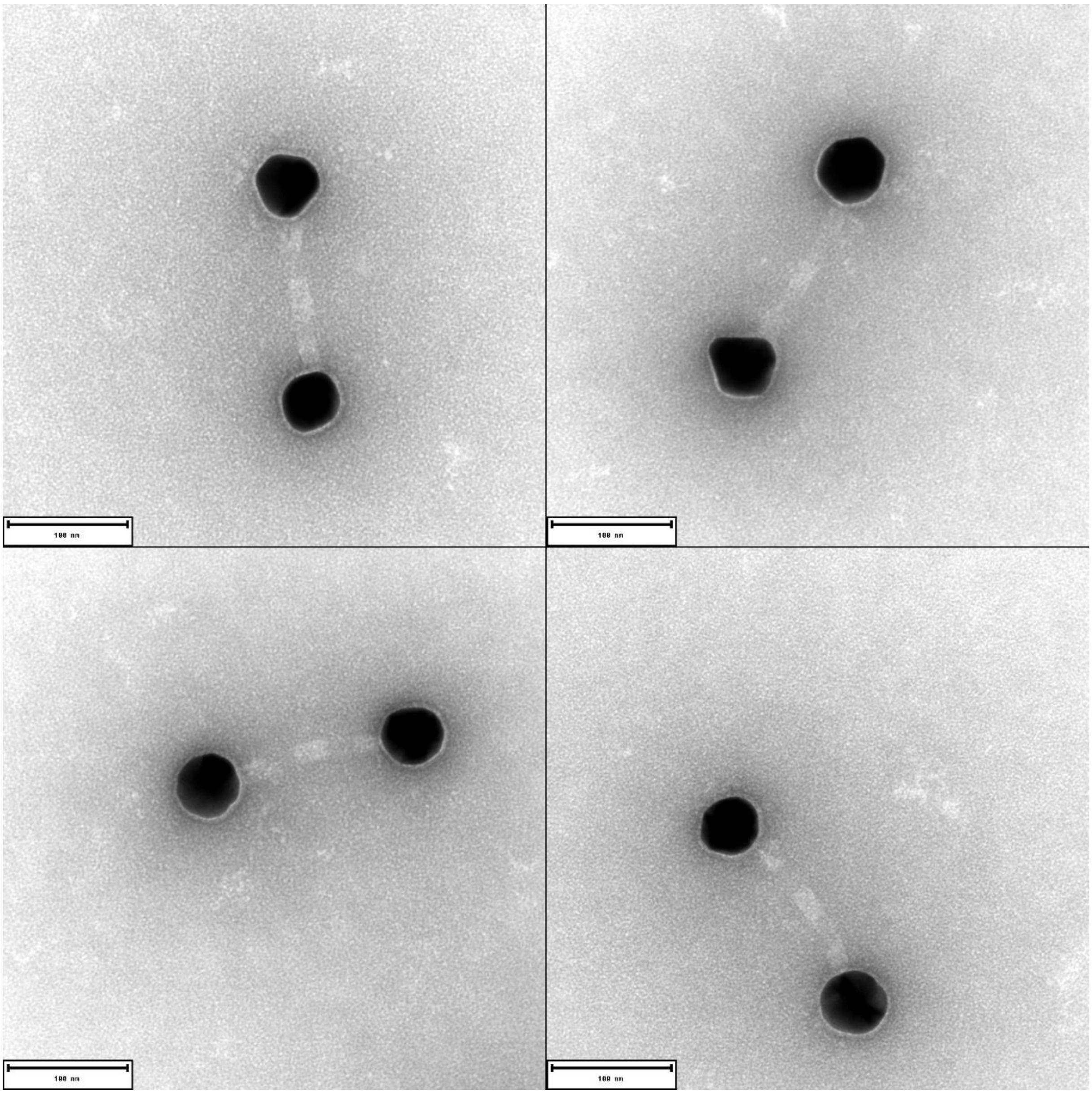

Figure S15. Exemplary TEM micrographs of the switch with AuNPs in the open state. Scale bars: 100 nm.

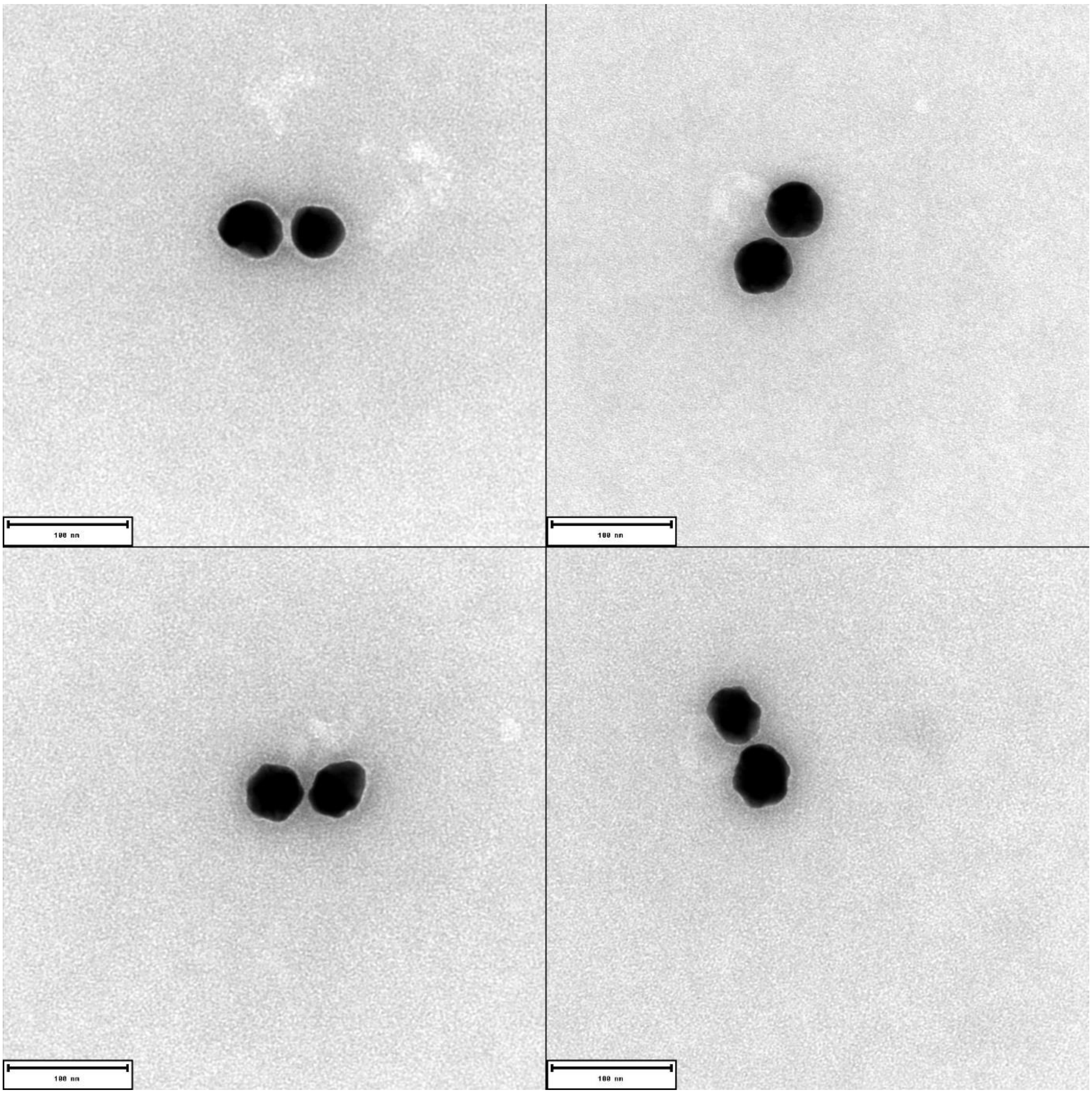

Figure S16. Exemplary TEM micrographs of the switch with AuNPs in the closed state. Scale bars: 100 nm.

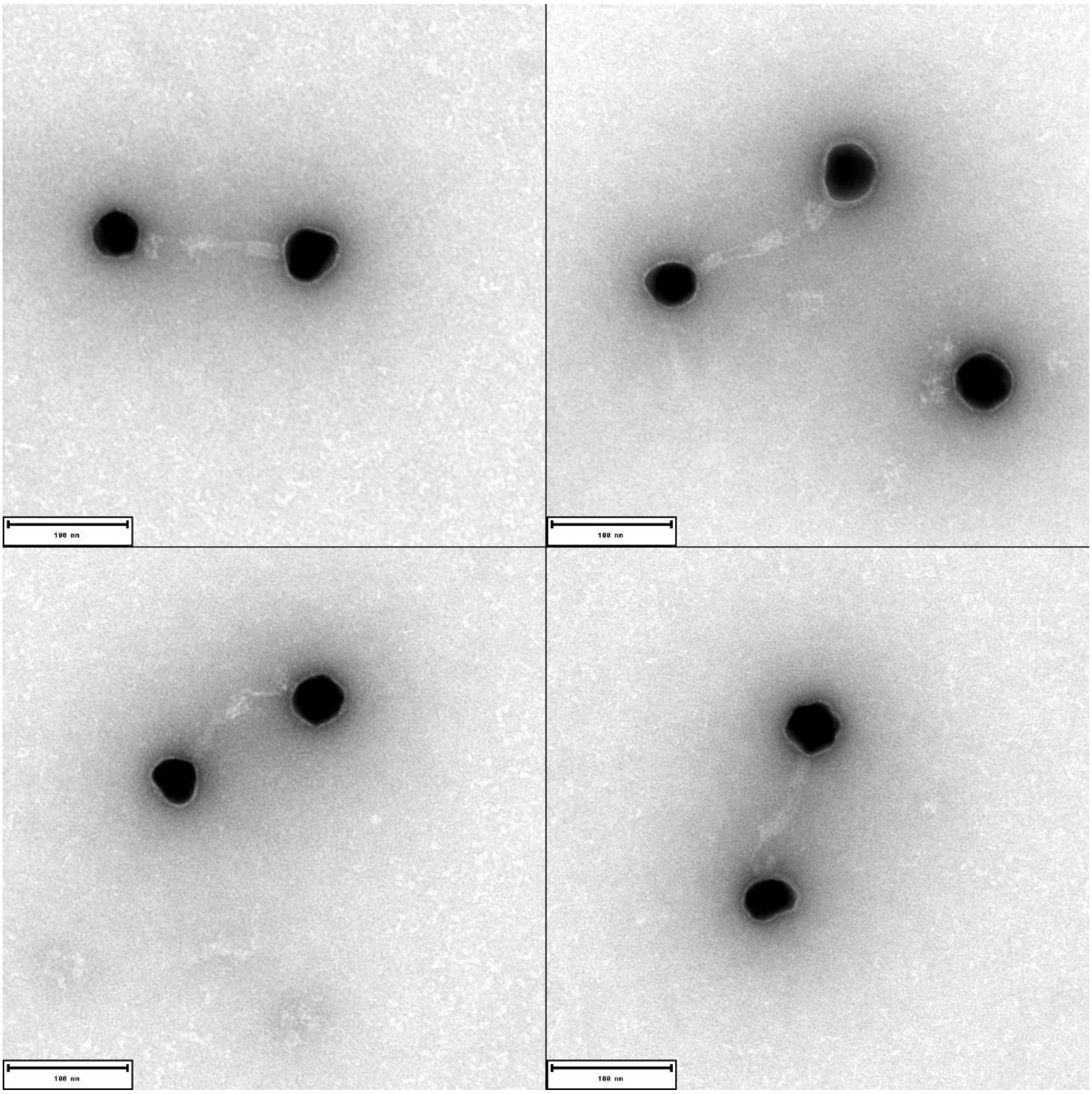

Figure S17. Exemplary TEM micrographs of the switch with AuNPs in the re-opened state. Scale bars: 100 nm.

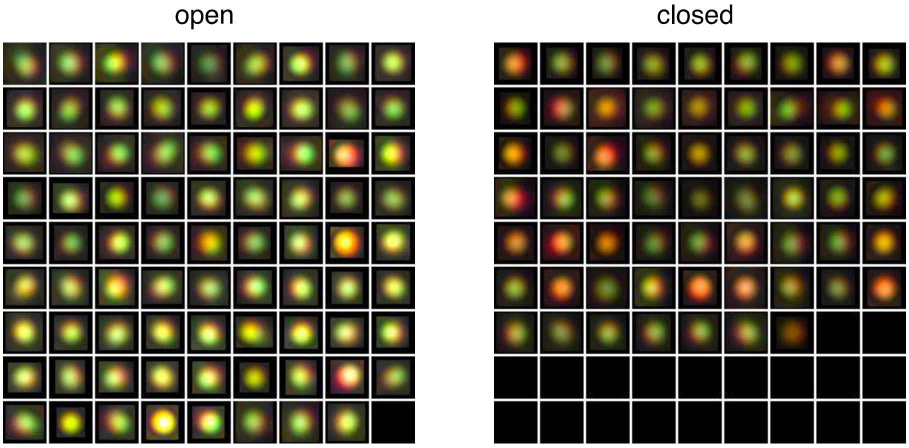

Figure S18. All analyzed single particles for the open state (left) and closed state (right) that were used for the RGB intensity distribution plot in Figure 3D in the main text.

Supporting Information Text S1: Entropic force of the ssDNA-segments

We modeled the ssDNA-segments inside the structure with a modified freely-jointed chain (mFJC) model. Specifically, we used the force-extension behavior of ssDNA described by Smith et al.[38]:

$$d(F) = L_C * \left[\coth\left(\frac{F l_K}{k_B T}\right) - \frac{k_B T}{F l_K}\right] * \left(1 + \frac{F}{S}\right)$$

with S being the stretch modulus and the contour length L_C being the number of nucleotides N of the ssDNA times the length per single base L_B:

$$L_C = N * L_B$$

We used the following parameters to calculate the resulting entropic force for a fixed end-to-end distance d (d is given by the structure design as described in Figures S1 and S2):

Parameter	Value
L_B	6.3 Å $\pm$ 0.8 Å
l_K	1.5 nm
S	800 pN
$k_B T$	4.114 pNnm
d	29.92 nm

The resulting force for each individual ssDNA-segment was calculated to be 5.56 pN. Since we have always two parallel ssDNA-segments connecting the three blocks in our design (see figs. S1 and S2 for design schematics) we can multiply the calculated force per individual ssDNA-segment by a factor of two. This gives us an effective force of around 11 pN acting on each of the three blocks pulling them towards each other.

Table S1: nonlinear thermal annealing ramp for DNA origami nanostructures

Temperature (°C)	Time (min)	Temperature (°C)	Time (min)
65	5	44	75
64-61	3	43	60
60-59	15	42	45
58	30	41-39	30
57	45	38-37	15
56	60	36-30	8
55	75	29-25	2
54-45	90	4	storage

Table S2: List of oligonucleotides used for the DNA origami nanostructures

Name	Sequence	color
Core	AGTATTACGAAGGTGTTTGTTCGCCTAAAAGGG	
Core	TAGCTTCAAATATCGAGGATTAGAGACCAATT	
Core	CGCGAGAAAGGAAGGGAAGCGATTTAGAAAAC	
Core	ACCGATCGCTGAGGCCGATTAAGAATAGCGGAACAAGCACCC	
Core	TTTCTTGTATCTCGGAATCGCCATTAAAAATGCTGAGAG	
Core	AAGCCCGAAAGACTAAAGAACTCTAAAGTCTG	
Core	GTTGAAAATCTCCAAAGGTGAATGGCGC	
Core	AAAAAGAAAGGAACAACTAATGCTAAACAAATG	
Core	TATTGCTGGGAAGACTTCTTACTGCTCAAACA	
Core	TAAGTCTTTACCCTGATTTTGTAAACTATCAGAGC	
Core	CAGGGAGTCGAGGGTAGCCCACG	
Core	GAGTATTACCTGTTATGTAAAAG	
Core	ATCAAAAATCAGATCAATCAGAATCGCGGGGACCATCAATGAAAAGA	
Core	CAGCATCGGAATAAAGGCCAAATCAAAAAGGGATTAAGGGCGAAGCT	
Core	CTGGTTTGTGGGATTTAGGAATTGTCCACAGATCAGAGCGCCCA	
Core	TGTGCCTTTACATAAATGAAGCGGCAGAGCAG	
Core	TCATTGAATCCCCCTGACCATAA	
Core	GCGGTAACGGCTACAAATACGTAGAAGGCACCAACCTACTCA	
Core	TTAGAGTCCATTGCTGTGAAGGTTTATAAGTAATCCTTT	
Core	GCAATGCATGACGACGGTAACACCCCATTCTGCAATGTG	
Core	ACCCTGTAATACTTTTGAGCATAACTCGCTACTCAAT	
Core	CGCCAGGGTGGTTTGAGAGAGTGGAAGCATTTTC	
Core	GTTATATTCCAATACCGTTAAGACCCG	
Core	CGTGAGCAAAAAGATAGAAGGAGCGCCAGG	
Core	CAGAGGGGGTAATAGTAACAATCCTCGCACAAGCCCCAACGCCAT	
Core	GGAGAGGCGGTTTGCGTACGAGCCTGCAGCAA	
Core	TTTCCTGTGTGAAATTGTTAGAGGACTAAAAGA	
Core	CAATTCCACACAACATATACTACATGCCTGGG	
Core	TCGGGTTTCAAAGGAGAGTGAG	
Core	TCCTGTTTGATGGTGAGAGCCACAGTC	
Core	TGTAAGCCCAATTGTAGCATTAAG	
Core	AATAAAGTGTATAGGTGTAGGCGGATATCCTCAAGAGA	
Core	TAGTAACTTTCATTCACCGCCTGGCCCTTTCTTTTC	
Core	ATAACGCTTTAGAGATCGGTTTTTAAAGAAA	
Core	GAGCAAACGGTTGATATACAGGCCGGTTGTACCAAATAT	
Core	ACATTCTTATCTGGTCAGTTGGATCTAGAT	
Core	TAGGGCGCTGGAGCACTAAGTGAACCATGGCTTTTAACGGGGTGCCGC	
Core	CCAGCAGACAATAACGGATCGCATTCACCCATTCAGCCAAATCAA	
Core	AGTGCCACACCGAACGACCAGTAAATCCGCTAACAGTAG	
Core	AACAGTTGATTCGTAATTGCTGAGGTATCGC	
Core	TGACCCTTCATCAAGAGTAATCTTGCCGAAAGAGGCTATCAGG	
Core	TTTTTGGGGTCGAGGTGCCGTAACAAGTGTAGCTTTCCTCTCC	
Core	TTACCTTTAATTTCGCCATTCCTCAT	

Core	CGTACAGATGGAACTGAACTAGCACCATTACC	
Core	GCGATGGGGAGCTAAACAGGAGGCTTTTCAC	
Core	CTACATCGGAACAAGCGCAGTCTCTGAATTCACAAACAGCATTG	
Core	CTAATAGATGTGAAAATGACCGTATTTTGGAA	
Core	TCGCATTGGCCTCAGGAAGAGATCCTTCTAAGAACGC	
Core	CAGGCTGTAGCCACTTGCCTGAGTAGTATTTTCAACATCCAA	
Core	GCCAGGAACCCTAAACAGTTAATGCCATTTTCGGAATCA	
Core	CCGTCAATTCGTATTACTAATGAAAGGCACTG	
Core	TATGATATCGAGGTGTAGATGGGGCTTATC	
Core	TCACCATGTTATTGTGTCGCCACCGTCACCGACTTGA	
Core	CGTTATTACATTCGCATTAGTAACTTGGAGGTCAAGCACTGAAACCAG	
Core	GCCCGAAGTTTGGATTATACATTATATTTTAATG	
Core	GGAGACAGTCAAATCATTTATTTGAACCCTCACATTATG	
Core	CTCAGGAGGGATAGCACCCCAGCAAGGGTTGACGAATAATAATTTTG	
Core	GGAAAAGCCTGGATCGGCCAACGCGCGG	
Core	AGGATCTGGAACTTATTAACTCTCGACAAATCCATCGAT	
Core	ATTAAATGCAAAAATAATAGGTC	
Core	ATATATTTTAAATGCAATTTTTATATCTGAAAGCTAAATAAGGCAAA	
Core	TCTTCCTCAGAACTGATAAACTTAGCCGAAAATCACCAGTGTA	
Core	CAGCGGGGGTTTTGCTCAGTACCATCACCGTA	
Core	AGGAACCGGAACTCATAGCCCCCTTATTTCATCGGCCCCTG	
Core	GGCGTCGACAACAGATAATATCAGATGATGCTGAATACGAGGA	
Core	ACGTTGGCACAATTACCGCGCCCAATAGCAA	
Core	ATAATGGGAATAACCTTGCATCATAGGTCTTGGGTTAT	
Core	CCTACTGGTAAGTGTTGTTCCAGTTTCCGAGATGGCGAAAA	
Core	TTGCGAGAAATAGAATCTGCCACTCTTCTGA	
Core	AAGATTCAAGGAATTATCAACCGTTCTAGCTATGTACCCCAAGAGAA	
Core	CTTTGACCTGCTTTTTCAGGTTTAGTACCGCCACTGACCCC	
Core	TTTCAGGTTTAACGTCCGGGAGAACAAATGAA	
Core	TATACCAGTTATTACAATATAATGTCATTGCCTGAGAAT	
Core	AGCTTTATTATTTCAAAACATAGCGATAGCAATTTATC	
Core	GTCACGACGTTGTACGTTAAATAAGAATAACGTGTGAT	
Core	CGATTAAGTTGGGTTACTAGAAAAAGCCT	
Core	CCAGCTGGCGAAAGTTATCAACAATAGATAAATAAACA	
Core	GGGCGATCGGTGCGAATAATATCCCGAACCTCC	
Core	TAAGAACTGGAGGGTTTTGAAGCCTTAACAATTTTA	
Core	CTTTAATCATTGTGAACACCCTGAACAAAGACGGGAGA	
Core	GTAAATTGGGCTTCGCTAATATCAGAGA	
Core	GCTTGCCCTGACGATAAAGGTGGCAACATAATTACGCA	
Core	AGACAAGAACCGGTCGATAGCAGCACCGAGCGTCAGCCAA	
Core	AAATTCTGTAAATCGTCGCTATTCCC	
Core	CAATAGTGTTAGATTACATCATTGTCATGCGTCCTGCAAC	
Core	CAGGTATCATATGCGTTATACAAATTCTGAA	
Core	CAGCTAATCAAGATGAACAACATCAGGACGTTCCATAT	
Core	ATTAACTGTACAGAGAGAATAACATGAAAATA	

Core	ATTAGCAAGGCCGTAAGTTTATTTCCAGCGCC	
Core	AGTTTGCCTTTTAATCAGTAGGAGTGTAGAATGGACCTAAAGG	
Core	GTATAAAGCCAACGCTCAACGTAAAGTAATTAGCTAATG	
Core	CGGTATGAATCTTACCTGCCAGTT	
Core	GGTAATAAGAGCAAGAAACAATGAAATAAA	
Core	GAACTGGCGCAAACGTAGACCCACAAGAATTGAGTTAAGC	
Core	GCGCGTTTAGCGTTTGAAATCCGCAGCATTATACCAAGC	
Core	GCTTAGGTGAGAGACTAGGCTATAGAATTACAACTAACAA	
Core	AAACCAAGAATCAATAATCGGCTGCGAGCATGT	
Core	GCCTAATTAACGCTAAACGCCAAATCAGTTGAGCTAT	
Core	ACCGATTGAAATTCATATGGTTTATGTCACAAT	
Core	TATATGTAAATGCTGATTGTCGGACGTGT	
Core	AATTTAGGCAGAGGCACCGACAAAAGAGTAGGGCTTAATTGAG	
Core	AGCCAGTAATACCGCACTCATCGAGCGGGTATT	
Core	GTAAGCAGATAGCCGAACAAAGTTCGGAATACCCAAGCAATAG	
Core	GGAAACCGAAGGGAGGGAAGGTAAAGACATTCA	
Core	TTTTCATAATCAAAATCTTAGGATTATTTCGGAACCTATT	
**Name**	**Sequence**	**color**
End staple	TTTTCAGCAAAATCAAAACTCGTGTTGAGCTTGAAATTTT	
End staple	TTTTAGATACATTTCGCAAAATTTAGTTTGACCATTTTTT	
End staple	TTTTTTGTATCGGTTTATCAGCTTGCTTTCGAAAAAAGGCGTTAGAACAGCCCTCACAAC	
End staple	TTTTGCAACGAAGTCCGTGAAGACGGCACTGGTGACCTGGAAGAGTTTTT	
End staple	GGAAACTGCAACGAGCGCAGAAGATAAAACAGAGGTGTTTT	
End staple	TTTTGATTGCATCAAAAAGAAGTCAGAAGCAAAGCGTTTT	
End staple	TTTTCGCCCACGCATGGGATCGTCACTTTT	
End staple	TTTTACATCAAACGCCGCGAGAAAGAAACCAGCAATTTTT	
End staple	TAGCATTATTTGGGGCGCGAGCTGAAAAGGTGGCATTTT	
End staple	TTTTTCAATTCTACTAAAATTAAGCATTTT	
End staple	TTTTGGAGTGAGAATGGAGCCTTTAATTTT	
End staple	TTTTTTCTGCGGCAGTTAATCGAACTAGCTGAAACGACATACATTGTTTT	
End staple	CTGCTGGATGAACGGCCAGGAGATTCGCGTCGTCTTCACAGCTTTT	
End staple	TTTTTTCAGAAAACGAGAATCAAATGCTTTAAACAGTTTT	
End staple	GAATTAGCAATAGTAGTCGATGAACGGTAATCGTATTTT	
End staple	TTTTCCTCAGCAGCGAAGACTTTTTCTTTT	
End staple	TTTTACCTTTTCCATGAATTTGGGGATTTGACGCAGTTTT	
End staple	TTTTATAAAGCCTCAGCGGGAGAAGCCTTTTT	
End staple	TTTTGCTGATTGCCCACAGTTTCAGCTTTT	
End staple	TTTTCAAGGAGTTTATATGTGCAGATTTT	
End staple	TTTTCTGGATAGCGTCCAATACTGCGGAATCGTCATAAAAAGTTTTGC	
End staple	TTTTATGAGGAAGTTTCCATTAAACGGGTAAGAGGCTTTTCCGCTCA	
End staple	ACCAGTGAGACGGGCAACATTTT	
End staple	TTTTTCGAAGTGAGCGAAATCAGTAAACAGAGAGGTTTTT	
End staple	TTTTTTATTTCAACGCAAGGATAAAAATGCCTGGAAAGGCC	
End staple	TTTTCCAGCTGCATTAATGAGGTGCCTAATGAGTGA	
End staple	GCATACAAATCAATGTAATGAGTATCAATGAG	

End staple	TAAACCCAATATTACATAAAATGTTTAGATTTT	
End staple	TTTTGATGCCAGAGTCTGTAGTGCATTTGAGGATTTAGAAGTTTTT	
End staple	CATGATAAATTCTACAAAGGATTTAGGAATACCACATTTTTT	
End staple	TTTTAAACTAGCATGGGCGGGTTTTGT	
End staple	TTTTAATATTTAAATTGTAAGAAGATTGTATAAGCATTTT	
End staple	TTTTCAAAATCCCTTATGCTTTTGCAACCGATATATTCGGTAGTTG	
End staple	ATTGGCAGATTCACCAAGGAATTGAGGAAGGTTATTTTT	
End staple	TCTTTGATTAGTAATAACATTCAACATGAAGTTTCATTGGGAAGAAAAATCTACGTTTT	
End staple	CGAGCACGTATAACGTGCGGTCACGCTGCGCGTTTT	
End staple	TTTTTGCGCGAAAGCATCACCTTGCTTTT	
End staple	TTTTCAGACCGGAAGCAAACTCCTGGTAATATCCAGAAC	
End staple	GGTCCGTTTTAATTCGAGCTTCAAAGCGAACTTTT	
End staple	TTTTGCGAACGTGGCCGACAATGACAACAACCATTTTT	
End staple	AAATCTAACTGATAGCGAGATAGAACCCTTCT	
End staple	TTTTAGGCGGTCAGTATTAACACCGATTAACCAACAGTTCAGGGTTTT	
End staple	TTTTAAAGTACGGTGTCTGGTTTTAAATATGCAACTTTTT	
End staple	TCAAGGCCAACACCTAAAACTGGGAGTAAGCGTATTGCTAAACTGGAAATTTT	
End staple	TTTTTCATTTTTGCGGATGGCTTGGCCTTGCAACA	
End staple	TTTTGACTCCAACGTCATTAGACAGGAACGGTACGCC	
End staple	TTTTCTAAAATATCTTTAGGAGCGCGCAACACAGCAATAAAATTTT	
End staple	TTTTGCTATTTTTGAGAGATAATGCCGGAGAGGGTATTTT	
End staple	TTTTTCATTTTTTAACCAATAGGAAAAACAGACGTTAATACTATTATTTAAGAGG	
End staple	TTTTAACCGCCACCCTCGTTCCGAAATCGGTTTT	
End staple	TTTTATTAGACTTTACAAACAATATCCGATAGATGAACGAAGTTTT	
End staple	TTTTAGATTCAAAAGGGTGAAGTAATGTGTAGGTAATTTT	
End staple	TTTTTTATCATTTTGCGGAACAAAGAAACCACCTGGGC	
End staple	TTTTGTAGCCAGCTTTCATCAAC	
End staple	TTTTAGAATACACTAAAACAAAACGAAAGAGGCAAATTTT	
End staple	TTTTACGCCTGGTCGTTCAGGGTGCAAATCCAACCTCCG	
End staple	TTTTCAGACGACGATAAAAACCATTATCCCATTTCCAGA	
End staple	TTTTAAGAGGCTGAGACAGTGCCGTCGAGAGGGTTGTTTT	
End staple	TTTTCAATTCATCAATATAAGATTATCAGATGATGGTTTT	
End staple	TATCATAACCCTCGTTTACTTTT	
End staple	TTTTTTTGTATCATCGCCCGCCACCCTCAGTTTT	
End staple	ATTCTGAAACCGCCACCAGAGCCACCACCGGAACCGCCTCCCTTTTT	
End staple	TTTTATGCGCCGCCTGAACCACCACCTTTTTAGAAGAGT	
End staple	TTTTCAACTAATGCAGATACATACGAGCGTCATTTTGCACC	
End staple	TTTTAACAGTGCCCGTAATGTACCGGCCC	
End staple	TTTTAATTATTTGCACGTAAAACAGAAACAATGGGTTCAA	
End staple	TTTTGTTTGAGGCTAATTTATCTTTCCTTATTCAGA	
End staple	TTTTACGGTCAATCATAATTAAAGAACGTGTTTT	
End staple	TTTTTAAGCGTCATACATCACCCAATACAAACTATAGTTAGACGT	
End staple	TTTTACAGTACCTTTTACATAGATGAAT	
End staple	TTTTTGCCGGAAACCAGGCACCGGCACCGCTTCTGGTTTT	
End staple	TTTTATAGGCTGGCTGAGGGGAAAGCCGTTTT	

End staple	AACGTAACAAAGCTACACCACGGAAGAAACGTCACCAAACAGACCAGGCGCTTTT	
End staple	TTTTCAGACGATTGGCCTTGATATTTACCGTTCCAGTTTT	
End staple	TTTTCAGAGGCGAATTATTCATTTTAATTAATTTCAATTACCTGAGTTTT	
End staple	GAGGCGTTTTAGCATCGGACGACGAACCGTGCATCTGCCATTTT	
End staple	TTTTCACCAGAACCATTGAGGCAGGTTTTT	
End staple	TTTTCAAAAGAAGATGATGAAATATAAATCAACAAACATCAAGAAATTTT	
End staple	TATAGAAGCGCATCGTACAGTATCAAATTTTTGTTAAATCAGCTTTT	
End staple	AAAAATTAGAGCCAGCGAACGAGGCGCAGTTTT	
End staple	GCAAACATGATTGACCGATTCTCCGTGGGAACTTTT	
End staple	TTTTCAGAGCCGCCACTCAGAGCCGCTTTT	
End staple	TTTTACAAAATTAATTACATTTAACAATTTCATATATTCCTTCCTGATT	
End staple	GGAAATTATTCATTAAAAGTACAACGGAGATTTT	
End staple	TTAGAATCCTTGGCCTGATTGCTTTGAATACCAAGTTACAAAATCGCGTTTT	
End staple	ACAGGAGGCCACCAGACAGTGCCTTGAGTTTTT	
End staple	AATCGCCACTTTTTCAAATTTTAATGGTTTTACCA	
End staple	CTATCTTACCGAAGCCCAACGTCAAAAATAAAAACAG	
End staple	CTGCGAACGAGTAGTGGTCAATAACCTGTT	
End staple	GAGCCCCAAAGCGAAAGGAGCGTTCTTAAACAGCTTGAT	
End staple	CAGTTGAAGTCACACGAACCACCAAGCGTG	
End staple	CACTAGGGAACCAACGGTGTTGAAACCAATATTCATTACCCAAATC	
End staple	GCGAAACAAGGTGAATTATCATC	
End staple	GTTGTGATTGTGCAAGTTGGAATTATCATCTTGAATTACCTTAC	
End staple	TTTTAAACGGCGTCCAAGAAAACAAGCAAGCCGTTTTT	
End staple	ATAGTAAGAGAGGCTTTTGCAGCGAGTAACAATCGTAGGAATCAA	
End staple	TTTTGATGATACAGCGACAGCAAATAAATCCTCATTAA	
End staple	TTTTATTAATGAAAGATGGCAGAAGACGCTGAAACGACGGCCAGTGCCA	
End staple	ACTCCAGTGCGGGAGCTGCGCAACTGTTGGGAA	
End staple	TTTTTTAATAAAACGAACTAACGTAGTTGCTAATTACCTTATGCGATTT	
End staple	GTTTAAACGCGCCTGTGGGGATGTGCTGCAAGG	
End staple	GATAAAAATACATACAGAAACACCAGAACGAGTA	
End staple	AAATAAGGCATCTTCTGACCTAAAATATTTTAGTTAATTTAATCGCA	
End staple	ATACCGACACACCGGAATCATAATAACGCCAGGGTTTTCCCA	
End staple	ACATGTTCCTGTCCAGACGACGACTAAGAGAATATAAAGTATTTTCG	
End staple	AGAAACCAGTCCTGAACAAGAAAGGCCTCTTCGCTATTACG	
End staple	CCAAAAGCGCATTAGTCAGAGGGTAATTGAGGAGATGGTTTAATTTCAA	
End staple	GTATGTTAATGATTAAGACTCCTTGGAAACGCAATAATAAACCAGAA	
End staple	CAATAGATAAAAGAAACGCAAAGGCTCATTCAGTGAATAAG	
End staple	GCAGCCTTATCCAAATAAGAAACGATTTTTTGTTTTTTTTAAGAAAA	
End staple	GCCATTTGGGGACAAAAGGGCTATTGAC	
End staple	AGACAAAGAACGCGAGAAAATATTTAACAACGCCAACATGT	
**Name**	**Sequence**	**color**
Biotin handle	CATTCTCCTATTACTACCCGACTCCAGCTTTAAGCGCCATGCTCCTTTTGATAAGAGGTTTT	
Biotin handle	CATTCTCCTATTACTACCTAAGAACTGGAGGGTTTTGAAGCCTTAACAATTTTA	
Biotin handle	CATTCTCCTATTACTACCATTTTCACCCGTCGGTAATGGGATTCGCGTCTGGCCTTCCTTTTT	
Biotin handle	CATTCTCCTATTACTACCATAAACAGCCATATTATAAATAGCGAGCAACAC	

Handle sequence Biotin	CATTCTCCTATTACTACC	
Biotin sequence	GGTAGTAATAGGAGAAGAATGTT -Biotin-TEG	
**Name**	**Sequence**	**color**
Painthandle	TTTTTTTGTCGTCTTTCCAGCGTAACGATCTAAAGTATACATCTA	
Painthandle	TTTTCATCGCTATTACGGGGTATCGACATCATTACGATACATCTA	
Painthandle	TTTTTGAACCTCAAATAATACAGTAATACATCTA	
Painthandle	TCAAACCCTCAGGATGCAGGTGAGTATCTATACATCTA	
Painthandle	TTTTTTCGTCACCAGATCAAGTTTTTATACATCTA	
Painthandle	TTTTGCATATGATGTCTGACGCTGGATTTTAAAAGTTTGAGTAACAATACATCTA	
Painthandle	TTTTATATAAGTATATAACACTGAGTATACATCTA	
Painthandle	CGCCCTCAGAGCCACCACCCCCTCAGAACATGAAAGTATTATACATCTA	
Painthandle	GAAACAGTACATATGTGAGTAAGGGTTAGAACCTACCATATCAAATACATCTA	
Handle sequence Paint	ATA CAT CTA	
Imager sequence	C TAG ATG TAT - ATTO 647N	
**Name**	**Sequence**	**color**
AU mod 1	CCCCTTTGTCGTCTTTCCAGCGTAACGATCTAAAGTAAAAAAAAAAAAAAA	
AU mod 2	CCCCCATCGCTATTACGGGGTATCGACATCATTACGAAAAAAAAAAAAAAA	
AU mod 3	CCCCTGAACCTCAAATAATACAGTAAAAAAAAAAAAAAAA	
AU mod 4	TCAAACCCTCAGGATGCAGGTGAGTATCTAAAAAAAAAAAAAAA	
AU mod 5	CCCCTTCGTCACCAGATCAAGTCCCCAAAAAAAAAAAAAAA	
AU mod 6	CCCCGCATATGATGTCTGACGCTGGATTTTAAAAGTTTGAGTAACAAAAAAAAAAAAAAAA	
AU mod 7	CCCCATATAAGTATATAACACTGAGTAAAAAAAAAAAAAAA	
AU mod 8	CGCCCTCAGAGCCACCACCCCCTCAGAACATGAAAGTATTAAAAAAAAAAAAAAA	
AU mod 9	GAAACAGTACATATGTGAGTAAGGGTTAGAACCTACCATATCAAAAAAAAAAAAAAAAA	
**Name**	**Sequence**	**color**
DS	CGAGAAATGACTGATACCGTGC	
DS	AAAATTATTATCACGAGTACG	
DS	GTGGAAACGATACTTGCCCTCT	
DS	CTGTACAAAACATATAGATGAT	
DS	TTTATGGAGATGATAAATGCAC	
DS	TTCCGAGTCACAGGAGAATGG	
DS	ATCCCCGGGTACCGAGCTCGAA	
DS	TTCGTAATCATGGTCATAGCTG	
DS	TCGTCTGAAATGGATTATTTAC	
DS	TACCTACATTTTGACGCTCAA	
DS	ACAGGAAAAACGCTCATGGAAA	
DS	AATATTACCGCCAGCCATTGCA	
DS	AATTAACCGTTGTAGCAATACT	
DS	AAAGAGTCTGTCCATCACGCA	
DS	TAATCAGTGAGGCCACCGAGTA	
DS	AGAATCCTGAGAAGTGTTTTTA	
**Name**	**Sequence**	**color**
TH	TGGTATTCGAGAAATGACTGATACCGTGC	
TH	TGGTATTAAAATTATTATCACGAGTACG	
TH	TGGTATTGTGGAAACGATACTTGCCCTCT	

TH	TGGTATTCTGTACAAAACATATAGATGAT	
TH	TGGTATTTTTATGGAGATGATAAATGCAC	
TH	TGGTATTTTCCGAGTCACAGGAGAATGG	
TH	TGGTATTATCCCCGGGTACCGAGCTCGAA	
TH	TGGTATTTTCGTAATCATGGTCATAGCTG	
TH	TGGTATTTCGTCTGAAATGGATTATTTAC	
TH	TGGTATTTACCTACATTTTGACGCTCAA	
TH	TGGTATTACAGGAAAAACGCTCATGGAAA	
TH	TGGTATTAATATTACCGCCAGCCATTGCA	
TH	TGGTATTAATTAACCGTTGTAGCAATACT	
TH	TGGTATTAAAGAGTCTGTCCATCACGCA	
TH	TGGTATTTAATCAGTGAGGCCACCGAGTA	
TH	TGGTATTAGAATCCTGAGAAGTGTTTTTA	
**Name**	**Sequence**	**color**
SD	GCACGGTATCAGTCATTTCTCGAATACCA	
SD	CGTACTCGTGATAATAATTTTAATACCA	
SD	AGAGGGCAAGTATCGTTTCCACAATACCA	
SD	ATCATCTATATGTTTTGTACAGAATACCA	
SD	GTGCATTTATCATCTCCATAAAAATACCA	
SD	CCATTCTCCTGTGACTCGGAAAATACCA	
SD	TTCGAGCTCGGTACCCGGGGATAATACCA	
SD	CAGCTATGACCATGATTACGAAAATACCA	
SD	GTAAATAATCCATTTCAGACGAAATACCA	
SD	TTGAGCGTCAAAATGTAGGTAAATACCA	
SD	TTTCCATGAGCGTTTTTCCTGTAATACCA	
SD	TGCAATGGCTGGCGGTAATATTAATACCA	
SD	AGTATTGCTACAACGGTTAATTAATACCA	
SD	TGCGTGATGGACAGACTCTTTAATACCA	
SD	TACTCGGTGGCCTCACTGATTAAATACCA	
SD	TAAAAACACTTCTCAGGATTCTAATACCA	
**Name**	**Sequence**	**color**
Toehold sequence handle	TGGTATT	
Au handle sequence	AAAAAAAAAAAAAAA	
Strand displacement sequence	AATACCA	

Scaffold sequence:

segment 1:
CAGCTATGACCATGATTACGAATTCGAGCTCGGTACCCGGGGATCCATTCTCCTGTGACTCGGAAGTGCATTTAT
CATCTCCATAAA

segment 2:
ATCATCTATATGTTTTGTACAGAGAGGGCAAGTATCGTTTCCACCGTACTCGTGATAATAATTTTGCACGGTATC
AGTCATTTCTCG

segment 3:
GTAAATAATCCATTTCAGACGATTGAGCGTCAAAATGTAGGTATTTCCATGAGCGTTTTTCCTGTTGCAATGGCT
GGCGGTAATATT

segment 4:
AGTATTGCTACAACGGTTAATTTGCGTGATGGACAGACTCTTTTACTCGGTGGCCTCACTGATTATAAAAACACT
TCTCAGGATTCT

M13 hairpin 1:
GGCGGGTGTGGTGGTTACGCGCAGCGTGACCGCTACACTTGCCAGCGCCCTAGCGCCCGCTCCTTTCGCTTTC

M13 hairpin 2:
GGGTGATGGTTCACGTAGTGGGCCATCGCCC

Full p8634 scaffold sequence with segments and hairpins highlighted:

TGATAGACGGTTTTTCGCCCTTTGACGTTGGAGTCCACGTTCTTTAATAGTGGACTCTTGTTCCAAACTGGAACAACACTCAA
CCCTATCTCGGGCTATTCTTTTGATTTATAAGGGATTTTGCCGATTTCGGAACCACCATCAAACAGGATTTTCGCCTGCTGGG
GCAAACCAGCGTGGACCGCTTGCTGCAACTCTCTCAGGGCCAGGCGGTGAAGGGCAATCAGCTGTTGCCCGTCTCACTGGTGA
AAAGAAAAACCACCCTGGCGCCCAATACGCAAACCGCCTCTCCCCGCGCGTTGGCCGATTCATTAATGCAGCTGGCACGACAG
GTTTCCCGACTGGAAAGCGGGCAGTGAGCGCAACGCAATTAATGTGAGTTAGCTCACTCATTAGGCACCCCAGGCTTTACACT
TTATGCTTCCGGCTCGTATGTTGTGTGGAATTGTGAGCGGATAACAATTTCACACAGGAAACAGCTATGACCATGATTACGAA
TTCGAGCTCGGTACCCGGGGATCCATTCTCCTGTGACTCGGAAGTGCATTTATCATCTCCATAAAACAAAACCCGCCGTAGCG
AGTTCAGATAAAATAAATCCCCGCGAGTGCGAGGATTGTTATGTAATATTGGGTTTAATCATCTATATGTTTTGTACAGAGAG
GGCAAGTATCGTTTCCACCGTACTCGTGATAATAATTTTGCACGGTATCAGTCATTTCTCGCACATTGCAGAATGGGGATTTG
TCTTCATTAGACTTATAAACCTTCATGGAATATTTGTATGCCGACTCTATATCTATACCTTCATCTACATAAACACCTTCGTG
ATGTCTGCATGGAGACAAGACACCGGATCTGCACAACATTGATAACGCCCAATCTTTTTGCTCAGACTCTAACTCATTGATAC
TCATTTATAAACTCCTTGCAATGTATGTCGTTTCAGCTAAACGGTATCAGCAATGTTTATGTAAAGAAACAGTAAGATAATAC
TCAACCCGATGTTTGAGTACGGTCATCATCTGACACTACAGACTCTGGCATCGCTGTGAAGACGACGCGAAATTCAGCATTTT
CACAAGCGTTATCTTTTACAAAACCGATCTCACTCTCCTTTGATGCGAATGCCAGCGTCAGACATCATATGCAGATACTCACC
TGCATCCTGAACCCATTGACCTCCAACCCCGTAATAGCGATGCGTAATGATGTCGATAGTTACTAACGGGTCTTGTTCGATTA
ACTGCCGCAGAAACTCTTCCAGGTCACCAGTGCAGTGCTTGATAACAGGAGTCTTCCCAGGATGGCGAACAACAAGAAACTGG
TTTCCGTCTTCACGGACTTCGTTGCTTTCCAGTTTAGCAATACGCTTACTCCCATCCGAGATAACACCTTCGTAATACTCACG
CTGCTCGTTGAGTTTTGATTTTGCTGTTTCAAGCTCAACACGCAGTTTCCCTACTGTTAGCGCAATATCCTCGTTCTCCTGGT
CGCGGCGTTTGATGTATTGCTGGTTTCTTTCCCGTTCATCCAGCAGTTCCAGCACAATCGATGGTGTTACCAATTCATGGAAA
AGGTCTGCGTCAAATCCCCAGTCGTCATGCATTGCCTGCTCTGCCGCTTCACGCAGTGCCTGAGAGTTAATTTCGCTCACTTC
GAACCTCTCTGTTTACTGATAAGTTCCAGATCCTCCTGGCAACTTGCACAAGTCCGACAACCCTGAACGACCAGGCGTCTTCG
TTCATCTATCGGATCGCCACACTCACAACAATGAGTGGCAGATATAGCCTGGTGGTTCAGGCGGCGCATTTTTATTGCTGTGT
TGCGCTGTAATTCTTCTATTTCTGATGCTGAATCAATGATGTCTGCCATCTTTCATTAATCCCTGAACTGTTGGTTAATACGC
ATGAGGGTGAATGCGAATAATAAAGCTTGGCACTGGCCGTCGTTTTACAACGTCGTGACTGGGAAAACCCTGGCGTTACCCAA
CTTAATCGCCTTGCAGCACATCCCCCTTTCGCCAGCTGGCGTAATAGCGAAGAGGCCCGCACCGATCGCCCTTCCCAACAGTT
GCGCAGCCTGAATGGCGAATGGCGCTTTGCCTGGTTTCCGGCACCAGAAGCGGTGCCGGAAAGCTGGCTGGAGTGCGATCTTC
CTGAGGCCGATACTGTCGTCGTCCCCTCAAACTGGCAGATGCACGGTTACGATGCGCCCATCTACACCAACGTGACCTATCCC
ATTACGGTCAATCCGCCGTTTGTTCCCACGGAGAATCCGACGGGTTGTTACTCGCTCACATTTAATGTTGATGAAAGCTGGCT
ACAGGAAGGCCAGACGCGAATTATTTTTGATGGCGTTCCTATTGGTTAAAAAATGAGCTGATTTAACAAAAATTTAATGCGAA
TTTTAACAAAATATTAACGTTTACAATTTAAATATTTGCTTATACAATCTTCCTGTTTTTGGGGCTTTTCTGATTATCAACCG
GGGTACATATGATTGACATGCTAGTTTTACGATTACCGTTCATCGATTCTCTTGTTTGCTCCAGACTCTCAGGCAATGACCTG
ATAGCCTTTGTAGATCTCTCAAAAATAGCTACCCTCTCCGGCATTAATTTATCAGCTAGAACGGTTGAATATCATATTGATGG
TGATTTGACTGTCTCCGGCCTTTCTCACCCTTTTGAATCTTTACCTACACATTACTCAGGCATTGCATTTAAAATATATGAGG
GTTCTAAAAATTTTTATCCTTGCGTTGAAATAAAGGCTTCTCCCGCAAAAGTATTACAGGGTCATAATGTTTTTGGTACAACC
GATTTAGCTTTATGCTCTGAGGCTTTATTGCTTAATTTTGCTAATTCTTTGCCTTGCCTGTATGATTTATTGGATGTTAATGC
TACTACTATTAGTAGAATTGATGCCACCTTTTCAGCTCGCGCCCCAAATGAAAATATAGCTAAACAGGTTATTGACCATTTGC
GAAATGTATCTAATGGTCAAACTAAATCTACTCGTTCGCAGAATTGGGAATCAACTGTTATATGGAATGAAACTTCCAGACAC
CGTACTTTAGTTGCATATTTAAAACATGTTGAGCTACAGCATTATATTCAGCAATTAAGCTCTAAGCCATCCGCAAAAATGAC
CTCTTATCAAAAGGAGCAATTAAAGGTACTCTCTAATCCTGACCTGTTGGAGTTTGCTTCCGGTCTGGTTCGCTTTGAAGCTC
GAATTAAAACGCGATATTTGAAGTCTTTCGGGCTTCCTCTTAATCTTTTTGATGCAATCCGCTTTGCTTCTGACTATAATAGT

CAGGGTAAAGACCTGATTTTTGATTTATGGTCATTCTCGTTTTCTGAACTGTTTAAAGCATTTGAGGGGGATTCAATGAATAT
TTATGACGATTCCGCAGTATTGGACGCTATCCAGTCTAAACATTTTACTATTACCCCCTCTGGCAAAACTTCTTTTGCAAAAG
CCTCTCGCTATTTTGGTTTTTATCGTCGTCTGGTAAACGAGGGTTATGATAGTGTTGCTCTTACTATGCCTCGTAATTCCTTT
TGGCGTTATGTATCTGCATTAGTTGAATGTGGTATTCCTAAATCTCAACTGATGAATCTTTCTACCTGTAATAATGTTGTTCC
GTTAGTTCGTTTTATTAACGTAGATTTTTCTTCCCAACGTCCTGACTGGTATAATGAGCCAGTTCTTAAAATCGCATAAGGTA
ATTCACAATGATTAAAGTTGAAATTAAACCATCTCAAGCCCAATTTACTACTCGTTCTGGTGTTTCTCGTCAGGGCAAGCCTT
ATTCACTGAATGAGCAGCTTTGTTACGTTGATTTGGGTAATGAATATCCGGTTCTTGTCAAGATTACTCTTGATGAAGGTCAG
CCAGCCTATGCGCCTGGTCTGTACACCGTTCATCTGTCCTCTTTCAAAGTTGGTCAGTTCGGTTCCCTTATGATTGACCGTCT
GCGCCTCGTTCCGGCTAAGTAACATGGAGCAGGTCGCGGATTTCGACACAATTTATCAGGCGATGATACAAATCTCCGTTGTA
CTTTGTTTCGCGCTTGGTATAATCGCTGGGGGTCAAAGATGAGTGTTTTAGTGTATTCTTTTGCCTCTTTCGTTTTAGGTTGG
TGCCTTCGTAGTGGCATTACGTATTTTACCCGTTTAATGGAAACTTCCTCATGAAAAAGTCTTTAGTCCTCAAAGCCTCTGTA
GCCGTTGCTACCCTCGTTCCGATGCTGTCTTTCGCTGCTGAGGGTGACGATCCCGCAAAAGCGGCCTTTAACTCCCTGCAAGC
CTCAGCGACCGAATATATCGGTTATGCGTGGGCGATGGTTGTTGTCATTGTCGGCGCAACTATCGGTATCAAGCTGTTTAAGA
AATTCACCTCGAAAGCAAGCTGATAAACCGATACAATTAAAGGCTCCTTTTGGAGCCTTTTTTTTGGAGATTTTCAACGTGAA
AAAATTATTATTCGCAATTCCTTTAGTTGTTCCTTTCTATTCTCACTCCGCTGAAACTGTTGAAAGTTGTTTAGCAAAATCCC
ATACAGAAAATTCATTTACTAACGTCTGGAAAGACGACAAAACTTTAGATCGTTACGCTAACTATGAGGGCTGTCTGTGGAAT
GCTACAGGCGTTGTAGTTTGTACTGGTGACGAAACTCAGTGTTACGGTACATGGGTTCCTATTGGGCTTGCTATCCCTGAAAA
TGAGGGTGGTGGCTCTGAGGGTGGCGGTTCTGAGGGTGGCGGTTCTGAGGGTGGCGGTACTAAACCTCCTGAGTACGGTGATA
CACCTATTCCGGGCTATACTTATATCAACCCTCTCGACGGCACTTATCCGCCTGGTACTGAGCAAAACCCCGCTAATCCTAAT
CCTTCTCTTGAGGAGTCTCAGCCTCTTAATACTTTCATGTTTCAGAATAATAGGTTCCGAAATAGGCAGGGGGCATTAACTGT
TTATACGGGCACTGTTACTCAAGGCACTGACCCCGTTAAAACTTATTACCAGTACACTCCTGTATCATCAAAAGCCATGTATG
ACGCTTACTGGAACGGTAAATTCAGAGACTGCGCTTTCCATTCTGGCTTTAATGAGGATTTATTTGTTTGTGAATATCAAGGC
CAATCGTCTGACCTGCCTCAACCTCCTGTCAATGCTGGCGGCGGCTCTGGTGGTGGTTCTGGTGGCGGCTCTGAGGGTGGTGG
CTCTGAGGGTGGCGGTTCTGAGGGTGGCGGCTCTGAGGGAGGCGGTTCCGGTGGTGGCTCTGGTTCCGGTGATTTTGATTATG
AAAAGATGGCAAACGCTAATAAGGGGGCTATGACCGAAAATGCCGATGAAAACGCGCTACAGTCTGACGCTAAAGGCAAACTT
GATTCTGTCGCTACTGATTACGGTGCTGCTATCGATGGTTTCATTGGTGACGTTTCCGGCCTTGCTAATGGTAATGGTGCTAC
TGGTGATTTTGCTGGCTCTAATTCCCAAATGGCTCAAGTCGGTGACGGTGATAATTCACCTTTAATGAATAATTTCCGTCAAT
ATTTACCTTCCCTCCCTCAATCGGTTGAATGTCGCCCTTTTGTCTTTGGCGCTGGTAAACCATATGAATTTTCTATTGATTGT
GACAAAATAAACTTATTCCGTGGTGTCTTTGCGTTTCTTTTATATGTTGCCACCTTTATGTATGTATTTTCTACGTTTGCTAA
CATACTGCGTAATAAGGAGTCTTAATCATGCCAGTTCTTTTGGGTATTCCGTTATTATTGCGTTTCCTCGGTTTCCTTCTGGT
AACTTTGTTCGGCTATCTGCTTACTTTTCTTAAAAAGGGCTTCGGTAAGATAGCTATTGCTATTTCATTGTTTCTTGCTCTTA
TTATTGGGCTTAACTCAATTCTTGTGGGTTATCTCTCTGATATTAGCGCTCAATTACCCTCTGACTTTGTTCAGGGTGTTCAG
TTAATTCTCCCGTCTAATGCGCTTCCCTGTTTTTATGTTATTCTCTCTGTAAAGGCTGCTATTTTCATTTTTGACGTTAAACA
AAAAATCGTTTCTTATTTGGATTGGGATAAATAATATGGCTGTTTATTTTGTAACTGGCAAATTAGGCTCTGGAAAGACGCTC
GTTAGCGTTGGTAAGATTCAGGATAAAATTGTAGCTGGGTGCAAAATAGCAACTAATCTTGATTTAAGGCTTCAAAACCTCCC
GCAAGTCGGGAGGTTCGCTAAAACGCCTCGCGTTCTTAGAATACCGGATAAGCCTTCTATATCTGATTTGCTTGCTATTGGGC
GCGGTAATGATTCCTACGATGAAAATAAAAACGGCTTGCTTGTTCTCGATGAGTGCGGTACTTGGTTTAATACCCGTTCTTGG
AATGATAAGGAAAGACAGCCGATTATTGATTGGTTTCTACATGCTCGTAAATTAGGATGGGATATTATTTTTCTTGTTCAGGA
CTTATCTATTGTTGATAAACAGGCGCGTTCTGCATTAGCTGAACATGTTGTTTATTGTCGTCGTCTGGACAGAATTACTTTAC
CTTTTGTCGGTACTTTATATTCTCTTATTACTGGCTCGAAAATGCCTCTGCCTAAATTACATGTTGGCGTTGTTAAATATGGC
GATTCTCAATTAAGCCCTACTGTTGAGCGTTGGCTTTATACTGGTAAGAATTTGTATAACGCATATGATACTAAACAGGCTTT
TTCTAGTAATTATGATTCCGGTGTTTATTCTTATTTAACGCCTTATTTATCACACGGTCGGTATTTCAAACCATTAAATTTAG
GTCAGAAGATGAAATTAACTAAAATATATTTGAAAAAGTTTTCTCGCGTTCTTTGTCTTGCGATTGGATTTGCATCAGCATTT
ACATATAGTTATATAACCCAACCTAAGCCGGAGGTTAAAAAGGTAGTCTCTCAGACCTATGATTTTGATAAATTCACTATTGA
CTCTTCTCAGCGTCTTAATCTAAGCTATCGCTATGTTTTCAAGGATTCTAAGGGAAAATTAATTAATAGCGACGATTTACAGA
AGCAAGGTTATTCACTCACATATATTGATTTATGTACTGTTTCCATTAAAAAAGGTAATTCAAATGAAATTGTTAAATGTAAT
TAATTTTGTTTTCTTGATGTTTGTTTCATCATCTTCTTTTGCTCAGGTAATTGAAATGAATAATTCGCCTCTGCGCGATTTTG
TAACTTGGTATTCAAAGCAATCAGGCGAATCCGTTATTGTTTCTCCCGATGTAAAAGGTACTGTTACTGTATATTCATCTGAC
GTTAAACCTGAAAATCTACGCAATTTCTTTATTTCTGTTTTACGTGCAAATAATTTTGATATGGTAGGTTCTAACCCTTCCAT
TATTCAGAAGTATAATCCAAACAATCAGGATTATATTGATGAATTGCCATCATCTGATAATCAGGAATATGATGATAATTCCG
CTCCTTCTGGTGGTTTCTTTGTTCCGCAAAATGATAATGTTACTCAAACTTTTAAAATTAATAACGTTCGGGCAAAGGATTTA
ATACGAGTTGTCGAATTGTTTGTAAAGTCTAATACTTCTAAATCCTCAAATGTATTATCTATTGACGGCTCTAATCTATTAGT
TGTTAGTGCTCCTAAAGATATTTTAGATAACCTTCCTCAATTCCTTTCAACTGTTGATTTGCCAACTGACCAGATATTGATTG
AGGGTTTGATATTTGAGGTTCAGCAAGGTGATGCTTTAGATTTTTCATTTGCTGCTGGCTCTCAGCGTGGCACTGTTGCAGGC
GGTGTTAATACTGACCGCCTCACCTCTGTTTTATCTTCTGCTGGTGGTTCGTTCGGTATTTTTAATGGCGATGTTTTAGGGCT
ATCAGTTCGCGCATTAAAGACTAATAGCCATTCAAAAATATTGTCTGTGCCACGTATTCTTACGCTTTCAGGTCAGAAGGGTT
CTATCTCTGTTGGCCAGAATGTCCCTTTTATTACTGGTCGTGTGACTGGTGAATCTGCCAATGTAAATAATCCATTTCAGACG
ATTGAGCGTCAAAATGTAGGTATTTCCATGAGCGTTTTTCCTGTTGCAATGGCTGGCGGTAATATTGTTCTGGATATTACCAG
CAAGGCCGATAGTTTGAGTTCTTCTACTCAGGCAAGTGATGTTATTACTAATCAAAGAAGTATTGCTACAACGGTTAATTTGC
GTGATGGACAGACTCTTTTACTCGGTGGCCTCACTGATTATAAAAACACTTCTCAGGATTCTGGCGTACCGTTCCTGTCTAAA
ATCCCTTTAATCGGCCTCCTGTTTAGCTCCCGCTCTGATTCTAACGAGGAAAGCACGTTATACGTGCTCGTCAAAGCAACCAT
AGTACGCGCCCTGTAGCGGCGCATTAAGCGCGGCGGGTGTGGTGGTTACGCGCAGCGTGACCGCTACACTTGCCAGCGCCCTA
GCGCCCGCTCCTTTCGCTTTCTTCCCTTCCTTTCTCGCCACGTTCGCCGGCTTTCCCCGTCAAGCTCTAAATCGGGGGCTCCC
TTTAGGGTTCCGATTTAGTGCTTTACGGCACCTCGACCCCAAAAAACTTGATTTGGGTGATGGTTCACGTAGTGGGCCATCGC
CC
